# Ferroptosis in the tumor immune microenvironment: from danger signals to immune reprogramming

**DOI:** 10.3389/fimmu.2026.1978294

**Published:** 2026-10-14

**Authors:** Qingmiao Shi, Bingqian Tan, Huiwu Xing

**Affiliations:** 1 The First Affiliated Hospital, College of Clinical Medicine, Henan University of Science and Technology, Luoyang, China; 2 Department of Hepatobiliary Surgery, Children's Hospital of Chongqing Medical University, National Clinical Research Center for Children and Adolescents' Health and Diseases, Ministry of Education Key Laboratory of Child Development and Disorders, Chongqing Key Laboratory of Pediatric Metabolism and Inflammatory Diseases, Chongqing, China; 3 The First Affiliated Hospital of Zhengzhou University, Zhengzhou, Henan, China

**Keywords:** ferroptosis, immune checkpoint blockade, lipid peroxidation, nanomedicine, tumor immune microenvironment

## Abstract

Immune-checkpoint blockade has changed cancer therapy, yet durable responses remain limited by immune exclusion, myeloid suppression and metabolic adaptation within the tumor immune microenvironment. Ferroptosis, an iron-dependent form of lipid-peroxidation-driven regulated cell death, offers a mechanistic bridge between tumor metabolism and antitumor immunity. Ferroptotic tumor cells can release danger signals, oxidized lipid mediators and antigenic material that shape dendritic-cell activation, macrophage behavior, myeloid-derived suppressor cell function and CD8+ T-cell responses. Based on currently heterogeneous evidence, we propose a working model in which acute and spatially restricted tumor-cell ferroptosis may favor immune priming and checkpoint sensitivity, whereas persistent or broadly distributed lipid-peroxidation stress may impair effector immune cells and sustain suppressive states. This temporal-spatial distinction remains incompletely validated in directly comparable experimental systems. This review summarizes the biochemical foundations of ferroptosis, the immune signals released by ferroptotic cells, the reprogramming of innate and adaptive immune compartments, and the emerging use of ferroptosis inducers, nanomedicines and immune-checkpoint combinations. We also discuss biomarkers, *in vivo* monitoring and immune-cell-specific toxicity as key barriers to clinical translation. A central conclusion is that ferroptosis should be treated not as a monolithic tumoricidal modality, but as a microenvironment-specific immune remodeling process whose therapeutic value depends on timing, localization and preservation of antitumor immune function.

## 1 Introduction

Immune-checkpoint inhibitors have created durable therapeutic benefit in several cancers by reinvigorating antitumor T-cell immunity, but response heterogeneity remains a defining feature of this treatment class (1, 2). Resistance to anti-programmed cell death protein 1 (PD-1) therapy and anti-programmed death-ligand 1 (PD-L1) therapy reflects coordinated changes in antigen presentation, interferon signaling, tumor-intrinsic survival programs and immune-cell states within the tumor microenvironment (3). Recent syntheses of immunotherapy resistance emphasize that physical exclusion, suppressive myeloid cells and metabolic competition can limit T-cell function even when a checkpoint target is present (4). Combination strategies therefore increasingly aim to reprogram the tumor immune microenvironment (TIME), rather than simply add another cytotoxic pressure to tumor cells (5).

PD-L1 regulation is itself embedded in oncogenic, inflammatory and stromal signaling, making checkpoint sensitivity partly dependent on the metabolic and immune state of the tissue niche (6). Tumor-associated macrophages (TAMs) are particularly important because they can restrain cytotoxic lymphocytes, shape PD-1/PD-L1 responses and determine whether local inflammation becomes antitumor or immunosuppressive (7). Broader reviews of immunotherapy biomarkers and combination therapy similarly identify the TIME as a major determinant of response, especially when tumor-cell intrinsic markers alone fail to predict clinical benefit (8). Strategies that target PD-1/PD-L1 within the microenvironment now include approaches directed at myeloid cells, stromal barriers and metabolic vulnerabilities (9). Neoadjuvant checkpoint blockade has further highlighted the value of studying immune remodeling while tumors remain in place, because local tissue microenvironment can reveal response mechanisms that are lost in late metastatic sampling (10). These developments create a strong rationale for reviewing cell-death programs that do more than remove malignant cells and can instead reshape immune signaling at the tumor site (11).

Ferroptosis has emerged as one such program because it couples iron metabolism, polyunsaturated lipid oxidation and antioxidant failure to immune-relevant tissue stress. Building on existing pathway- and therapy-oriented syntheses, this review adopts a temporal, spatial and cell-type-specific framework to interpret how ferroptosis reshapes the TIME. Temporally, we distinguish acute ferroptotic injury and transient danger signaling from persistent lipid-peroxidation stress and chronic immune dysfunction. Spatially, we consider whether ferroptotic stress is restricted to malignant tissue or broadly distributed across the tumor microenvironment and immune compartments. At the cellular level, we distinguish ferroptosis in tumor cells, suppressive myeloid populations and antitumor lymphocytes, because the same biochemical process may produce opposing immunological consequences depending on the affected cell type. This framework is used throughout the review to integrate ferroptotic danger signaling, innate and adaptive immune reprogramming, therapeutic combinations and translational barriers, with the aim of defining when, where and in which cells ferroptosis is likely to support rather than undermine antitumor immunity.

## 2 Ferroptosis and the immune tumor niche

Ferroptosis connects tumor metabolism to immune remodeling through a defined biochemical program and a set of tissue-level consequences. The pathway is governed by iron availability, phospholipid composition, lipid radical propagation and antioxidant systems that determine whether cells tolerate or succumb to oxidative membrane injury (**Figure 1**).

**Figure 1:**
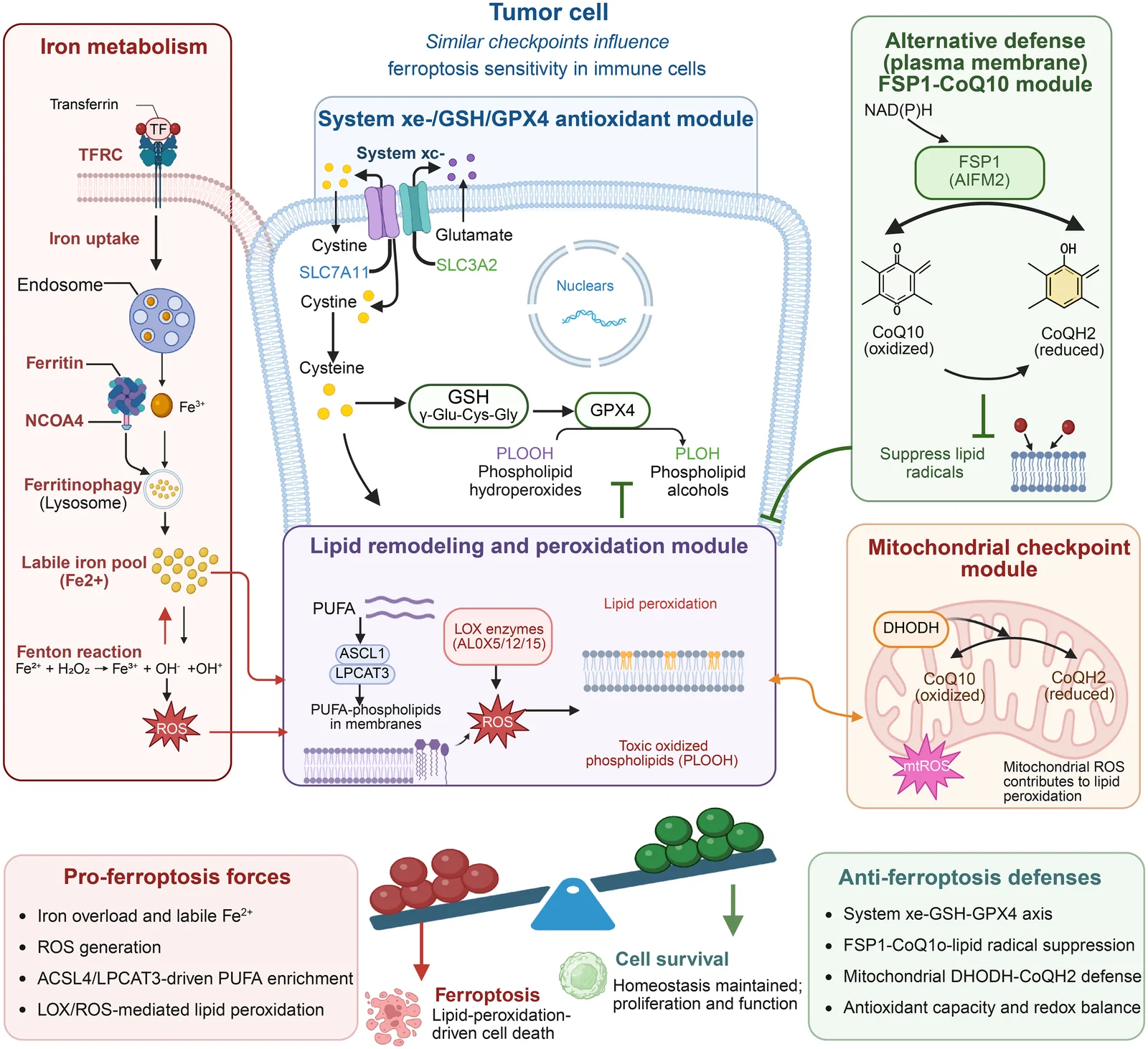
Ferroptosis pathways in the tumor immune microenvironment. The figure summarizes iron metabolism, GPX4-GSH defense, SLC7A11/system xc-, ACSL4/LPCAT3-dependent lipid remodeling, FSP1-CoQ10 defense and mitochondrial ferroptosis checkpoints. These mechanisms determine whether tumor and immune cells tolerate or undergo lipid-peroxidation-driven death.

### 2.1 Core ferroptosis pathways

Ferroptosis was defined as an iron-dependent, non-apoptotic cell-death process induced by small molecules and suppressed by iron chelation or lipid-reactive antioxidants (12, 13). Glutathione peroxidase 4 (GPX4) later emerged as a central suppressor of ferroptotic cancer-cell death because its inhibition permits toxic lipid peroxide accumulation (14). Acyl-CoA synthetase long-chain family member 4 (ACSL4) determines ferroptosis sensitivity by shaping the cellular pool of oxidizable phospholipids, thereby linking lipid metabolism to death susceptibility (15). Iron availability is also shaped by cellular iron-export pathways; in particular, SLC40A1 (ferroportin) regulates iron efflux, and dysregulation of this transporter can alter intracellular iron accumulation and thereby influence ferroptosis susceptibility (16). A broader redox framework positions ferroptosis at the intersection of metabolism, oxidative stress, membrane biology and disease pathogenesis (17, 18).

Cancer reviews have extended these mechanisms into tumor suppression, drug resistance and microenvironmental adaptation (19). Contemporary overviews also emphasize that ferroptosis is not a single linear pathway, because GPX4, system xc-, ferroptosis suppressor protein 1, dihydroorotate dehydrogenase and mitochondrial metabolism can create parallel buffering systems (20). These parallel systems matter for immunotherapy because a tumor may resist immune-induced ferroptosis through one antioxidant module while remaining vulnerable through another. Experimental assignment of ferroptosis nevertheless requires more than the detection of lipid peroxidation or altered expression of canonical regulators. Current methodological recommendations favor convergent evidence, including lipid-peroxidation-associated cell death together with pharmacological rescue by established ferroptosis inhibitors such as ferrostatin-1 or liproxstatin-1, evidence of iron dependence using iron chelation where appropriate, and genetic perturbation of ferroptosis-regulatory pathways (21). Changes in GPX4, SLC7A11, ACSL4 or lipid-reactive oxygen species alone should therefore be interpreted as evidence of ferroptosis-associated pathway activity rather than definitive proof of ferroptotic cell death. Where feasible, apoptosis, necroptosis and other regulated cell-death mechanisms should also be experimentally distinguished, particularly in studies using combination therapies that may activate several death programs simultaneously (22). In this review, we therefore distinguish studies providing functional or rescue-based validation of ferroptosis from those reporting primarily ferroptosis-associated molecular or oxidative changes.

### 2.2 Ferroptosis as immune stress

Ferroptosis differs from immunologically silent cell loss because oxidized membranes and damaged organelles can be sensed by neighboring immune and stromal cells. Emerging evidence indicates that ferroptosis can alter immune infiltration, antigen availability and inflammatory signaling, but the direction of this effect depends on tumor type and immune microenvironment (23). Metabolic regulation of ferroptosis within the tumor microenvironment further links nutrient availability, hypoxia, lipid metabolism and redox buffering to immune-cell function (24). Lipid metabolic reprogramming is therefore a shared language between ferroptotic tumor cells and immune cells that either clear, present or respond to damaged tumor material (25).

### 2.3 The TIME as a ferroptosis receiver

The TIME contains immune populations with different ferroptosis thresholds and different responses to lipid stress. Reviews of ferroptosis in tumor progression and immunotherapy describe a context-dependent pattern in which ferroptosis can promote immune activation, immune escape or therapy resistance depending on the receiving cell compartment (26). Immunometabolic remodeling can also drive ferroptosis resistance, creating a suppressive niche in which tumor cells and immune cells adapt to chronic oxidative pressure (27). Systematic syntheses of ferroptosis and tumor immunity support this view by showing that ferroptosis-related cues intersect with macrophages, dendritic cells, myeloid-derived suppressor cells and T cells rather than acting only within malignant cells (28). Nanomedicine-oriented reviews add that delivery context is important because the same ferroptosis inducer can have different immune effects when distributed broadly or concentrated in tumor tissue (29). Together, these observations define the three axes used in this review—time, anatomical distribution and cellular identity—as interdependent determinants of the immunological outcome of ferroptosis.

## 3 Ferroptotic danger signals

Ferroptotic tumor cells influence the TIME by exporting molecular information. This information includes classical danger-associated molecular patterns, antigenic material and lipid-peroxidation products that can act as immune modulators rather than inert by-products (**Figure 2**; **Table 1**).

**Figure 2:**
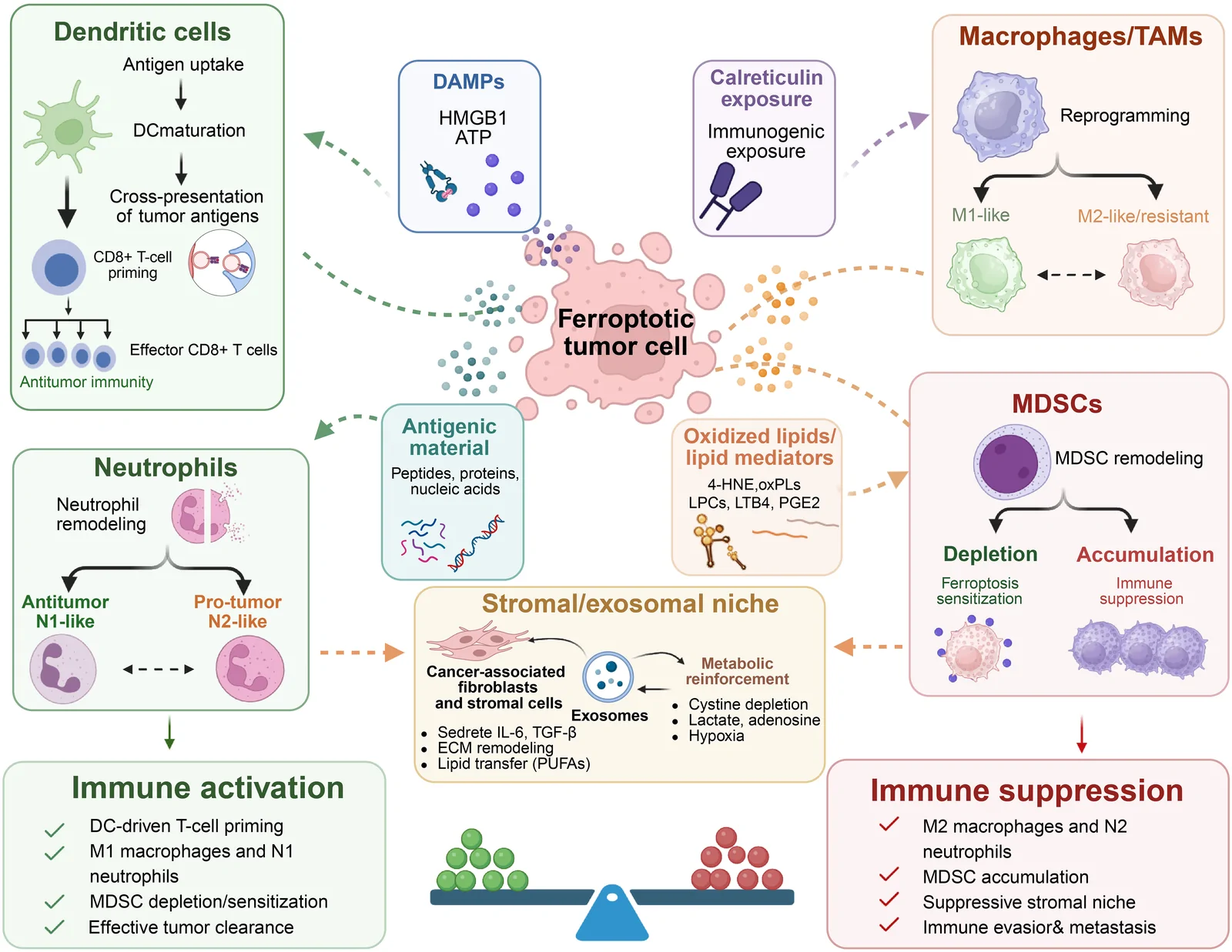
Ferroptosis-derived signals and innate immune remodeling. Ferroptotic tumor cells release danger-associated molecular patterns, oxidized lipids and antigenic material that can activate dendritic cells, repolarize macrophages, alter myeloid-derived suppressor cells and reshape neutrophil function.

**Table 1:** Core mechanisms linking ferroptosis to immune remodeling in cancer.

Cancer type	Immune compartment	Ferroptosis mediator	Main immune effect	Evidence basis	Reference
Pancreatic cancer	Mixed antitumor immunity	Tumor-specific GPX4 degradation	Initiated ferroptosis-linked antitumor immunity	Mouse tumor models	(30)
Cancer vaccine models	Dendritic-cell priming	Early ferroptotic tumor cells	Induced efficient antitumor immunity	Cell vaccination; mouse models	(31)
Pancreatic ductal adenocarcinoma	Antigen-presenting immunity	MCP-GPX4/HMGB1 signaling	Triggered immunogenic ferroptosis	Preclinical tumor models	(32)
Glioma	Dendritic cells	Ferroptosis-armed DC vaccine	Enhanced glioma immunotherapy	Vaccine model; mouse tumors	(33)
Lung adenocarcinoma	Macrophages and fibroblasts	IL4I1/TDO2-AhR metabolism	Drove immunosuppression and ferroptosis resistance	Patient-linked tumor analysis; models	(50)
Hepatocellular carcinoma	Macrophage-associated immunity	MerTK disruption	Enhanced ferroptosis and checkpoint response	Preclinical checkpoint model	(51)
Hepatocellular carcinoma	Immunosuppressive macrophages	Sorafenib plus FSP1 inhibition	Triggered dual tumor-myeloid ferroptosis	Mouse models	(52)
Hepatocellular carcinoma	TAMs	APOC1-related ferroptosis pathway	Promoted M2-to-M1 transition	Single-cell analysis; validation models	(53)
Liver tumors and metastases	MDSCs	Ferroptosis induction plus MDSC blockade	Sensitized tumors to checkpoint blockade	Mouse tumor models	(59)
Breast cancer	MDSCs	Caveolin-1-PKA-DRP1 signaling	Linked MDSC ferroptosis to immunosuppression	Preclinical models	(60)
Breast cancer	Neutrophils	Aconitate decarboxylase 1	Ferroptosis resistance promoted metastasis	Mouse and tumor models	(62)
Cancer models	Tumor neutrophils	Neutrophil ferroptosis	Generated immune suppression	Preclinical tumor models	(63)
Solid tumors	CD8+ T cells	IFN-gamma-SLC7A11 suppression	T cells induced tumor ferroptosis	Mouse and patient-linked models	(70)
Cancer models	CD8+ T cells and tumor cells	ACSL4-dependent lipid handling	Linked fatty acids to tumor ferroptosis	Mechanistic tumor models	(71)
Cancer models	Tumor cells under IFN-γ pressure	AGPAT3-dependent phospholipid remodeling	Increased susceptibility to IFN-γ-mediated ferroptosis; AGPAT3 has also been linked to cisplatin resistance and reduced apoptosis	Mechanistic ferroptosis models; chemotherapy-resistance study	(73, 77)
Cancer models	Tumor cells/antitumor immunity	USP8-mediated deubiquitination and GPX4 stabilization	Promoted ferroptosis resistance; USP8 has also been linked to cisplatin resistance through suppression of apoptosis	Mechanistic ferroptosis models; apoptosis/drug-resistance study	(74, 78)
Cancer models	Tumor cells under IFN-γ pressure	PSAT1-linked serine metabolism and GPX4 hydroxylation	Suppressed ferroptosis and immunotherapy response; PSAT1 has also been linked to EGFR-inhibitor resistance and apoptosis regulation	Mechanistic ferroptosis models; targeted-therapy resistance study	(75, 79)
Solid tumors	CD8+ T cells	CD36-mediated lipid uptake	Impaired CD8+ T-cell effector function	Immune-cell functional models	(82)
Cancer models	CD8+ T cells	PCIF1-regulated ferroptosis	Controlled CD8+ activation and immunity	Mouse and patient-linked models	(83)

### 3.1 DAMP release and immunogenic exposure

Tumor-specific GPX4 degradation in pancreatic cancer models enhanced ferroptosis-initiated antitumor immune responses, supporting the idea that ferroptotic death can create immune stimulation when tumor selectivity is achieved (30). Vaccination with early ferroptotic cancer cells induced antitumor immunity, indicating that the timing and state of ferroptotic death influence whether antigen presentation becomes productive (31). Targeting an MCP-GPX4/HMGB1 pathway in pancreatic ductal adenocarcinoma was reported to trigger immunogenic ferroptosis, linking GPX4 suppression, high mobility group box 1 signaling and immune activation in a tumor model (32). Ferroptosis-armed dendritic-cell vaccines in glioma further support the concept that ferroptotic programs can be engineered to enhance antigen presentation and antitumor immunity (33).

Several delivery-oriented studies show that danger signaling can be spatially programmed rather than left to occur passively after tumor-cell death. Irradiated microparticles suppressed prostate cancer by combining tumor microenvironment reprogramming with ferroptosis, supporting the use of tumor-derived material as an immune-redox intervention (34). A polymeric nanocarrier mediated immunogenic cell death through metabolic regulation and spatiotemporal orchestration, indicating that the timing of ferroptotic or oxidative stress can be built into immunotherapy design (35). A sonodynamic biomimetic nanoplatform targeted ferroptosis and CD47 as paired vulnerabilities, linking ferroptotic damage with removal of a phagocytosis checkpoint (36).

### 3.2 Dendritic-cell priming

Ferroptosis becomes immunologically useful only if damaged tumor material is converted into effective antigen presentation. Photothermal iron oxide nanoparticles induced immunogenic ferroptosis and supported synergistic colorectal cancer therapy, illustrating how ferroptosis can be coupled to immune activation by a delivery platform (37). These findings support a model in which ferroptosis is most immunogenic when oxidative tumor damage is paired with dendritic-cell activation rather than unmanaged tissue injury.

### 3.3 Lipid messengers

Lipid peroxidation is not only the execution step of ferroptosis, because oxidized lipids also influence immune-cell metabolism. Lipotoxicity, lipid peroxidation and ferroptosis have been framed as a therapeutic dilemma in cancer because the same oxidative lipid pressure can support tumor killing or immune dysfunction (38). This synthesis-level view justifies treating lipid-peroxidation products as signaling mediators, while the most direct CD8+ T-cell and tumor-evasion evidence is discussed in the adaptive-immunity section.

### 3.4 Temporal context and signal direction: a working model

Available evidence supports the context dependence of ferroptosis-associated immune responses rather than a uniformly immunostimulatory or immunosuppressive effect (39, 40). Previous studies and syntheses suggest that ferroptotic tumor-cell death can generate immunogenic signals under selected conditions (41, 42), whereas persistent lipid-peroxidation stress and ferroptosis within immune compartments may contribute to immunosuppressive states (43, 44). However, these observations arise from heterogeneous tumor models, cell populations, ferroptosis-induction methods and experimental time scales, and therefore do not directly establish a universal temporal transition from immune activation to immune suppression.

More recent primary studies further illustrate this context dependence. Ferroptotic tumor cells have been shown to impair dendritic-cell maturation, antigen cross-presentation and protective antitumor immunity, with the immunological outcome varying according to the stage of ferroptotic death (45). In addition, ferroptotic injury to tumor-associated dendritic cells can compromise T-cell priming and reduce treatment efficacy (46). These findings indicate that the immune consequences of ferroptosis depend not only on whether ferroptosis occurs, but also on the affected cellular compartment and the biological context in which lipid-peroxidation stress develops.

Accordingly, we regard the proposed distinction between acute, spatially restricted ferroptosis and persistent or broadly distributed lipid-peroxidation stress as a working model rather than an established rule. An important untested hypothesis is that the duration, anatomical distribution and cellular target of lipid-peroxidation stress jointly determine whether ferroptosis promotes productive immune activation or immune dysfunction. Direct validation will require longitudinal studies that manipulate ferroptosis duration and spatial distribution within the same experimental system while simultaneously tracking tumor and immune-cell states.

## 4 Innate immune reprogramming

Innate immune cells are major recipients of ferroptotic cues because ferroptosis can occur within immune cells as well as in tumor cells (47). Macrophages, dendritic cells, myeloid-derived suppressor cells and neutrophils differ in how they sense oxidized lipids, clear damaged cells and reshape immune suppression, making innate immunity a central site where ferroptosis changes from tumor-cell death into tissue-level immune remodeling (48).

### 4.1 Macrophage plasticity

Macrophage responses to ferroptosis are strongly context-dependent (49). IL4I1-positive macrophages and TDO2-positive myofibroblasts were associated with aryl hydrocarbon receptor-mediated immunosuppression and ferroptosis resistance in solid-predominant lung adenocarcinoma, linking macrophage-stromal metabolism to a resistant TIME (50). MerTK disruption enhanced checkpoint inhibitor efficacy by increasing ferroptosis and immune response in hepatocellular carcinoma, suggesting that macrophage efferocytosis or clearance programs can restrain productive ferroptotic immunity (51). Co-delivery of sorafenib and an FSP1 inhibitor triggered ferroptosis in tumor cells and immunosuppressive macrophages in hepatocellular carcinoma models, supporting dual targeting of malignant and myeloid antioxidant defenses (52). APOC1 inhibition promoted M2-to-M1 macrophage transition through a ferroptosis-related pathway and enhanced anti-PD-1 therapy in hepatocellular carcinoma, although the single-cell basis of this study requires careful mechanistic interpretation (53). Collectively, these papers argue against a universal M1-or-M2 conclusion and instead support a model in which macrophage ferroptosis biology depends on phagocytosis, antioxidant state and metabolic niche.

Additional studies broaden this macrophage-centered view beyond classical polarization. An itaconate transporter, SLC13A3, impaired tumor immunity by endowing ferroptosis resistance, linking immunometabolism to reduced ferroptotic vulnerability in the TIME (54). TYRO3 induced anti-PD-1/PD-L1 therapy resistance by limiting innate immunity and tumoral ferroptosis, indicating that receptor-tyrosine-kinase signaling can coordinate immune suppression with tumor-cell ferroptosis escape (55). Glycyrrhizic acid-loaded nanotherapy combined ferroptosis induction with HMGB1 blockade to enhance tumor immunotherapy, suggesting that danger signaling may need tuning rather than unrestricted amplification (56).

### 4.2 Dendritic cells and antigen presentation

Dendritic cells are the immune compartment most directly positioned to convert ferroptotic tumor material into T-cell priming. RSL3 treatment in HNSCC models was associated with ferroptosis-related tumor changes and increased immunogenic exposure, together with remodeling of the immune microenvironment (57). The remaining issue is not whether dendritic cells can respond to ferroptotic material, but which molecular patterns distinguish productive cross-presentation from inflammatory clearance.

The dendritic-cell arm can also become a site of immune failure when ferroptosis affects antigen-presenting cells themselves. Tim-3 facilitated dendritic-cell ferroptosis and impaired antitumor immunity in steatohepatitis-related hepatocellular carcinoma, showing that ferroptosis in immune cells can suppress rather than enhance antigen presentation (58). This finding reinforces the need to distinguish ferroptosis of tumor cells from ferroptosis of immune cells in mechanistic and therapeutic studies.

### 4.3 Myeloid-derived suppressor cells

Myeloid-derived suppressor cells (MDSCs) are a major barrier to checkpoint response in ferroptosis-based therapy. Combining ferroptosis induction with MDSC blockade sensitized primary liver tumors and metastases to immune-checkpoint blockade, providing direct evidence that ferroptosis induction may need myeloid suppression control to achieve durable immunity (59). Caveolin-1 was reported to drive MDSC ferroptosis through PKA-DRP1-mediated endoplasmic reticulum-mitochondria crosstalk and to shape breast cancer immunosuppression, indicating that MDSC ferroptosis can itself be an immune-regulatory event (60). Etomoxir enhanced cancer therapy by promoting imidazole ketone erastin-induced MDSC ferroptosis, further supporting the therapeutic logic of making suppressive myeloid cells ferroptosis-sensitive (61).

Together, these findings indicate that MDSCs occupy a distinct position in ferroptosis-based immunotherapy. Tumor-cell ferroptosis alone may be insufficient when suppressive myeloid populations remain intact, whereas increasing ferroptotic vulnerability within MDSCs may relieve an important barrier to antitumor immunity. Therefore, the therapeutic effect of ferroptosis induction may depend not only on tumor-cell death but also on whether the accompanying myeloid compartment is depleted or functionally reprogrammed.

### 4.4 Neutrophils

Neutrophils resisted ferroptosis and promoted breast cancer metastasis through aconitate decarboxylase 1, showing that ferroptosis resistance in a myeloid subset can support tumor progression (62). In contrast, ferroptosis of tumor neutrophils was reported to cause immune suppression in cancer, suggesting that neutrophil ferroptosis can either restrain or promote immunity depending on whether it removes suppressive cells or generates suppressive inflammatory products (63). This apparent tension makes neutrophils a useful example of why cell-type-specific ferroptosis thresholds are needed before systemic ferroptosis induction is translated clinically.

Therapeutic manipulation of neutrophil ferroptosis can reverse this logic in selected settings. Enhanced photodynamic therapy combined with inhibition of tumor neutrophil ferroptosis boosted anti-PD-1 therapy in gastric cancer, indicating that preservation or functional reshaping of neutrophils may be more useful than indiscriminate ferroptosis induction (64).

### 4.5 Stromal and metabolic niches

Ferroptosis also intersects with stromal and metabolic programs that tune innate immunity. Macrophage extracellular vesicles were reported to confer cancer ferroptosis resistance through PRDX6-mediated mitophagy inhibition, adding a vesicle-mediated mechanism to macrophage-tumor communication (65). The ISCU-p53 axis was linked to macrophage polarization and immunotherapy response in esophageal squamous cell carcinoma, connecting iron-sulfur biology to immune phenotype control (66). These studies show that ferroptosis-related immune remodeling is often distributed across tumor cells, macrophages, stromal cells and extracellular communication systems.

Hypoxic tumor exosomes suppressed macrophage inflammation and ferroptosis through NDUFV2, thereby enhancing bystander tumor radioresistance and adding hypoxia-dependent extracellular vesicles to the ferroptosis-resistance network (67). CD36-positive cancer-associated fibroblasts created an immunosuppressive hepatocellular carcinoma microenvironment through macrophage migration inhibitory factor secretion, linking lipid-handling stromal states to myeloid immune suppression (68). In low-grade glioma, NUAK2 was proposed as a ferroptosis-related vulnerability, suggesting that tumor lineage-specific kinases can shape how ferroptosis intersects with immune and metabolic states (69).

## 5 Adaptive immunity

Adaptive immunity gives ferroptosis its clearest double-edged character. CD8+ T cells can induce tumor ferroptosis through interferon signaling, but they can also be damaged by lipid overload and ferroptosis-like stress, creating a feedback loop that can support or undermine checkpoint therapy (**Figure 3**; **Table 1**).

**Figure 3:**
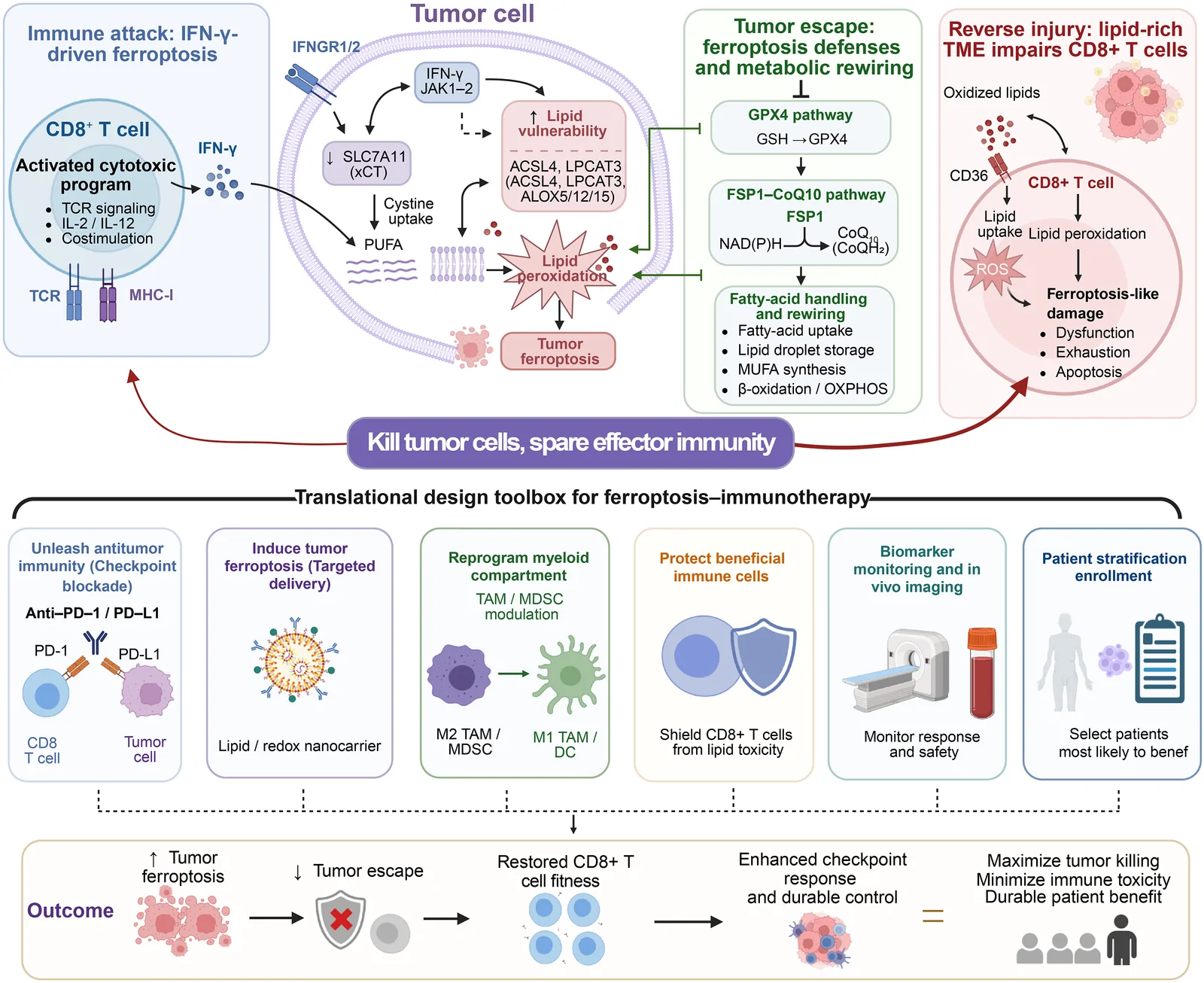
The CD8+ T-cell ferroptosis feedback loop. CD8+ T cells can promote tumor ferroptosis through interferon-gamma-mediated suppression of SLC7A11 and lipid remodeling, while oxidized lipid uptake can induce ferroptosis-like dysfunction in CD8+ T cells. The central message is that the same lipid-peroxidation biology can support tumor killing or impair effector immunity.

### 5.1 CD8+ T-cell-driven tumor ferroptosis

CD8+ T cells regulate tumor ferroptosis during cancer immunotherapy by producing interferon-gamma that suppresses SLC7A11 and increases tumor-cell vulnerability to lipid peroxidation (70). CD8+ T cells and fatty-acid metabolism also orchestrate tumor ferroptosis and immunity through ACSL4, which connects T-cell pressure with tumor lipid composition (71). Mefloquine enhanced anti-PD-1 efficacy through IFN-gamma-STAT1-IRF1-LPCAT3-induced tumor ferroptosis, extending the feedback loop to phospholipid remodeling downstream of interferon signaling (72). AGPAT3, an acylglycerol phosphate acyltransferase involved in membrane phospholipid remodeling, reshaped tumor-cell vulnerability to IFN-gamma-mediated ferroptosis and improved immunotherapy efficacy through lipid remodeling, further supporting the importance of membrane lipid composition in T-cell-induced tumor killing (73).

### 5.2 Escape from immune-induced ferroptosis

Tumor cells can evade this immune pressure by strengthening ferroptosis defense. USP8, a deubiquitinating enzyme involved in regulating protein stability, was reported to maintain GPX4 homeostasis and thereby modulate ferroptosis and cancer immunotherapy, identifying GPX4 stability as a resistance node (74). PSAT1, a phosphoserine aminotransferase involved in *de novo* serine biosynthesis and metabolic regulation, impaired ferroptosis and reduced immunotherapy efficacy through GPX4 hydroxylation, linking serine metabolism to GPX4-dependent immune resistance (75). Cancer cells avoided immune-induced ferroptosis via fatty-acid-binding proteins, indicating that lipid trafficking can protect tumor cells from T-cell-imposed oxidative pressure (76). These mechanisms convert ferroptosis from a death endpoint into a selectable resistance program under immune attack. These regulators can also be considered according to their broader biological functions and therapeutic relevance. AGPAT3 links membrane phospholipid remodeling to ferroptosis susceptibility; in other cancer contexts, AGPAT3 overexpression has also been associated with cisplatin resistance, increased cell survival, and reduced apoptosis in ovarian endometrioid carcinoma (77). USP8 connects deubiquitination-dependent protein stability with GPX4 homeostasis; beyond its role in ferroptosis, USP8 has also been implicated in cisplatin resistance by suppressing apoptosis in ovarian carcinoma (78). PSAT1 couples serine metabolism to GPX4-dependent antioxidant defense, while separate studies have shown that PSAT1 can promote EGFR-inhibitor resistance and tumor progression in lung adenocarcinoma, with PSAT1 depletion restoring drug sensitivity through ROS-dependent JNK/c-Jun-mediated apoptosis (79). These observations suggest that AGPAT3, USP8 and PSAT1 are better viewed as context-dependent determinants of cancer-cell survival and treatment response that intersect with ferroptosis rather than as ferroptosis-specific regulators. However, direct evidence that these factors broadly regulate additional forms of regulated cell death remains limited. Other tumor-intrinsic regulators extend this escape logic to lactate metabolism and death-receptor signaling. Apolipoprotein L3 enhanced CD8+ T-cell antitumor immunity in colorectal cancer and increased tumor-cell susceptibility to RSL3-associated ferroptotic stress through LDHA regulation, linking lactate metabolism to immune-sensitive ferroptosis (80). Tumor-cell-intrinsic Decr2 regulated ferroptosis and immunotherapy efficacy, indicating that peroxisomal lipid remodeling can influence checkpoint response through ferroptosis control (81). These studies support a view in which tumor-intrinsic ferroptosis regulators determine whether CD8+ T-cell pressure becomes durable tumor control or immune escape.

### 5.3 CD8+ T-cell ferroptosis and exhaustion

The same lipid stress that sensitizes tumor cells to ferroptosis can also compromise antitumor lymphocytes, making the cellular target of ferroptosis a critical determinant of its immunological outcome. CD36-mediated uptake of oxidized lipids promotes lipid peroxidation and ferroptosis in intratumoral CD8+ T cells, thereby impairing their effector function and antitumor activity (82). PCIF1 further regulates the ferroptotic susceptibility and activation state of CD8+ T cells, indicating that ferroptosis sensitivity in effector lymphocytes is controlled not only by extracellular lipid stress but also by cell-intrinsic regulatory programs (83). Together, these findings suggest that excessive ferroptotic stress within the CD8+ T-cell compartment may counteract the therapeutic benefit gained from inducing ferroptosis in tumor cells.

Importantly, this relationship differs across immune-cell populations. GPX4 prevents lipid peroxidation and ferroptosis in regulatory T cells (Tregs), thereby sustaining their activation and immunosuppressive activity (84). Thus, ferroptosis in effector CD8+ T cells may weaken antitumor immunity, whereas increased ferroptotic susceptibility of suppressive T-cell populations could potentially relieve immune suppression. These opposing effects illustrate that the balance between immune suppression and antitumor immunity is determined not by ferroptosis per se, but by which cellular compartment undergoes ferroptotic stress. Consistent with this principle, an albumin nanoassembly that bidirectionally manipulated ferroptosis in tumor cells and CD8+ T cells demonstrated a therapeutic strategy aimed at enhancing tumor-cell ferroptosis while limiting ferroptotic injury to effector lymphocytes (85).

Therapeutic interventions can therefore be viewed according to whether they shift this balance toward tumor-selective ferroptosis while maintaining immune competence. Intermittent dietary methionine deprivation facilitated tumoral ferroptosis and synergized with checkpoint blockade (86), A humanized CD36-blocking antibody unleashed antitumor immunity against liver cancer and liver metastasis, supporting CD36 blockade as a strategy to protect or restore lipid-stressed T-cell function (87). Icariin promoted mitochondrial dysfunction-associated ferroptosis in colorectal cancer and enhanced the efficacy of PD-1 inhibition (88). Although these tumor-directed interventions do not by themselves establish selective protection of immune cells, collectively they emphasize that effective ferroptosis-immunotherapy combinations should maximize ferroptotic vulnerability in malignant cells while avoiding detrimental lipid-peroxidation stress in antitumor lymphocytes.

## 6 Ferroptosis as an immune adjuvant

Ferroptosis-based therapy is most persuasive when it is designed as immune remodeling rather than nonspecific oxidative killing. The main translational aim is to sensitize tumors to checkpoint blockade while avoiding lipid injury to beneficial immune cells (**Figure 4**; **Table 2**).

**Figure 4:**
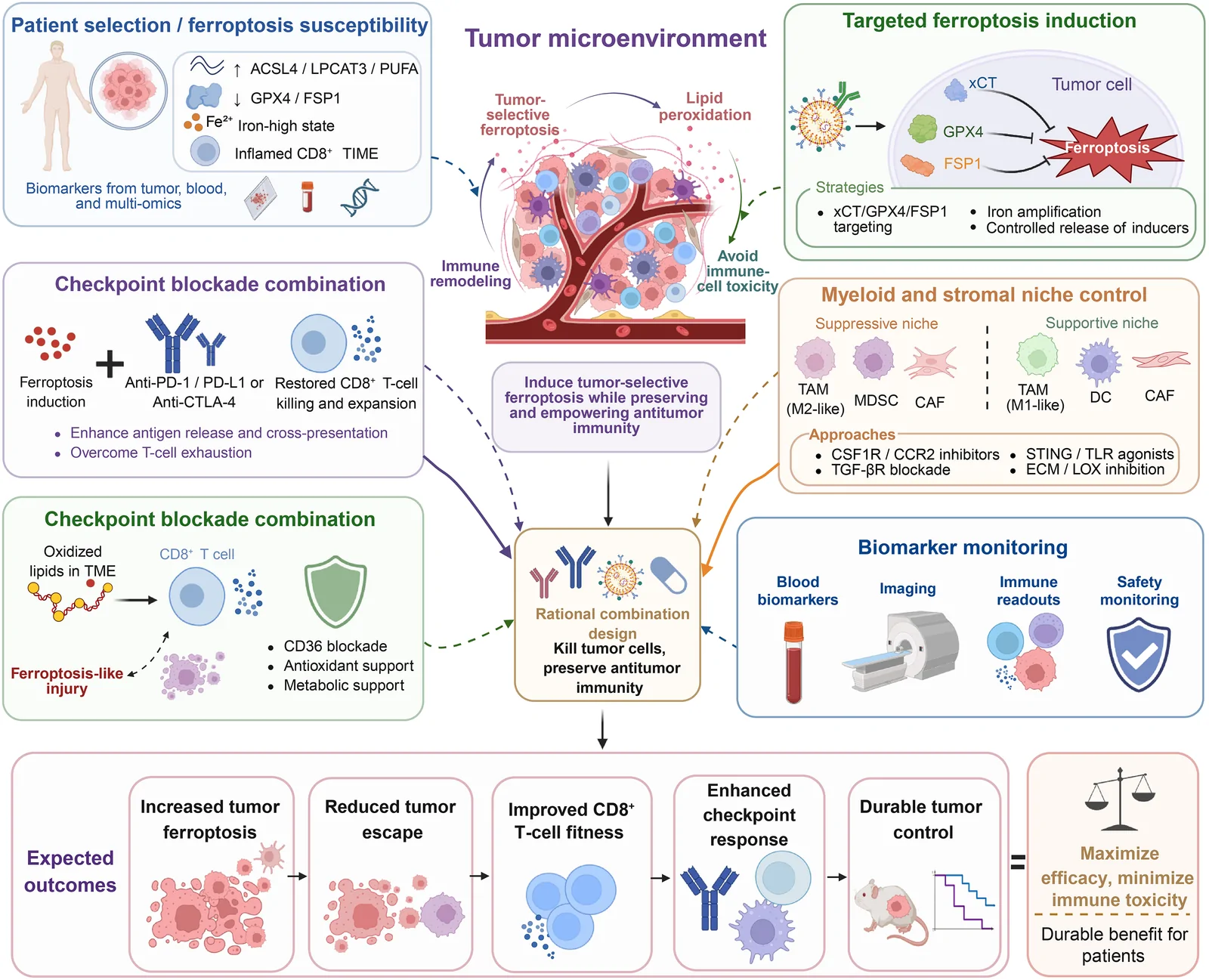
Translational design of ferroptosis-immunotherapy combinations. Rational therapy should align tumor ferroptosis susceptibility, targeted delivery, immune-checkpoint blockade, myeloid-state control, biomarker monitoring and immune-cell protection. This design reduces the risk that systemic ferroptosis induction will damage beneficial antitumor immunity.

**Table 2:** Ferroptosis-based strategies for cancer immunotherapy.

Cancer context	Strategy	Target or principle	Therapeutic relevance	Evidence basis	Reference
Lung cancer	CPT1A targeting	Fatty-acid oxidation-linked ferroptosis	Synergized with immunotherapy	Animal-model evidence	(90)
Colorectal cancer	CYP1B1 blockade	ACSL4 degradation prevention	Reversed anti-PD-1 resistance mechanism	Patient-associated evidence	(91)
Hepatocellular carcinoma	Matrix/PD-L2 modulation	SMYD3/xCT-linked ferroptotic susceptibility	Improved anti-PD-1 efficacy	Animal-model + patient-associated evidence	(93)
Cancer models	Kitasamycin	HUWE1-NCOA4-FTH1 pathway	Overcame ferroptosis and ICI resistance	Animal-model evidence	(94)
Melanoma	GBP2 condensate targeting	Ferroptosis sensitization	Increased checkpoint response	Animal-model evidence	(95)
Metastatic bladder cancer	Nano-immunotherapy	Ferroptosis plus STING activation	Combined redox injury with innate activation	Animal-model evidence	(101)
Bladder cancer	Mitochondrial liposome	Brequinar-driven mitochondrial ferroptosis	Promoted checkpoint blockade efficacy	Animal-model evidence	(102)
Lung cancer	NRF2 nanomodulator	Antioxidant defense suppression	Created immunostimulatory TIME	Animal-model evidence	(103)
Bladder cancer	Fe-EGCG@RSL3 nanoplatform	Ferroptosis and TIME remodeling	Enhanced bladder cancer immunotherapy	Animal-model evidence	(104)
Colorectal liver metastasis	Dual-targeting therapy	Tumor ferroptosis plus CAF reprogramming	Suppressed liver metastasis	Animal-model evidence	(108)
Melanoma	Nanofiber delivery	Local ferroptosis-associated oxidative stress	Stimulated antitumor immunity	Animal-model evidence	(109)
Lung cancer	PET-tracked codelivery	Ferroptosis and pyroptosis imaging	Supported monitored anti-PD-L1 synergy	Animal-model evidence	(118)
Cancer theranostics	Nanozyme platform	Catalytic ferroptosis induction	Supports imaging-guided translation	Review evidence	(119)
Cancer therapy	Magnetic nanomaterials	Imaging-compatible ferroptosis design	Enables theranostic development	Review evidence	(120)
Cancer models	Cell-specific targeting	Tumor killing with immune-cell sparing	Reduced immune-cell toxicity risk	Preclinical experimental evidence	(125)

Evidence levels are reported according to the highest level of evidence described in the cited study. “Patient-associated evidence” refers to analyses of human tumor specimens, patient-derived samples, or clinical datasets and should not be interpreted as evidence of prospective therapeutic efficacy. “Animal-model evidence” refers to therapeutic or mechanistic validation in experimental tumor models. Review evidence summarizes published preclinical or translational studies and does not constitute independent therapeutic validation. No strategy listed in this table should be interpreted as clinically validated unless prospective clinical evidence is specifically available.

### 6.1 Overcoming tumor-intrinsic ferroptosis resistance

A recurring mechanism underlying resistance to checkpoint-based ferroptosis therapy is the preservation of tumor-cell antioxidant and metabolic defenses. Accordingly, therapeutic sensitization can be achieved by lowering the ferroptotic threshold of tumor cells through several complementary routes. This sensitization principle also extends to targeted-therapy resistance. In hepatocellular carcinoma, AZ-628 enhanced the sensitivity of tumor cells to donafenib, with ferroptosis, apoptosis and epithelial–mesenchymal transition implicated in the reversal of drug resistance (89). Targeting carnitine palmitoyl transferase 1A induced ferroptosis and synergized with immunotherapy in lung cancer, linking fatty-acid oxidation to ferroptosis-based immune sensitization (90). CYP1B1 promoted anti-PD-1 resistance by facilitating ACSL4 degradation, whereas its inhibition restored a lipid-remodeling state more permissive to ferroptosis (91). More broadly, mechanical properties of the tumor microenvironment can generate spatially heterogeneous cellular states and treatment responses, highlighting tissue mechanics as an additional determinant of therapeutic susceptibility (92). Consistent with this principle, matrix stiffness-dependent PD-L2 deficiency increased susceptibility to xCT-associated ferroptotic stress and improved anti-PD-1 efficacy in hepatocellular carcinoma, illustrating how mechanical cues can regulate antioxidant defense and checkpoint sensitivity (93). Kitasamycin targeted the HUWE1-NCOA4-FTH1 pathway and thereby connected ferritinophagy-dependent iron regulation with reversal of ferroptosis and immunotherapy resistance (94). GBP2 condensates similarly promoted ferroptosis and sensitized melanoma to anti-PD-L1 therapy (95). Additional tumor-intrinsic resistance mechanisms converge on ferroptosis susceptibility through FSP1-dependent antioxidant defense, GPX4 regulation, ACSL4-dependent lipid remodeling, mitochondrial redox control, and metabolic adaptation. SLC6A8-mediated creatine uptake suppressed ERK2-FSP1 signaling and induced ferroptosis in colorectal cancer (96). GPX4 palmitoylation has been highlighted as a potential therapeutic target for enhancing antitumor immunity (97), whereas TMOD3 promoted immunotherapy resistance in KRAS-mutated pancreatic cancer through autophagy-dependent ACSL4 degradation (98). Vorapaxar enhanced mitochondria-associated ferroptosis through a FOXO1/HMOX1 mechanism (99). Simvastatin overcame pPCK1-pLDHA-SPRINGlac-mediated ferroptosis and chemo-immunotherapy resistance in AKT-hyperactivated intrahepatic cholangiocarcinoma, connecting metabolic lactylation-related resistance to ferroptosis restoration (100).

Collectively, these studies indicate that ferroptosis-associated checkpoint resistance is not governed by a single molecular pathway. Rather, lipid metabolism, cystine-dependent antioxidant defense, iron handling and other tumor-intrinsic stress-response programs can independently raise the threshold for ferroptotic death. Their therapeutic relevance therefore lies in restoring tumor-cell susceptibility to lipid-peroxidation stress imposed during antitumor immune responses, thereby creating a more permissive state for checkpoint blockade.

### 6.2 Spatial and subcellular control of ferroptosis induction

Nanomedicine may improve ferroptosis-immunotherapy not simply by delivering ferroptosis inducers, but by addressing three major limitations of systemic ferroptotic stress: insufficient tumor selectivity, lack of subcellular control and inadequate coordination with immune activation. Nano-immunotherapy combining ferroptosis with STING activation improved treatment of metastatic bladder cancer in preclinical models, illustrating how ferroptotic damage can be coupled directly to innate immune stimulation (101). Mitochondrial-targeted brequinar liposomes promoted mitochondrial ferroptosis and checkpoint-blockade immunotherapy, demonstrating that subcellular targeting may increase the precision of ferroptotic stress (102). An NRF2 nanomodulator induced lung-cancer ferroptosis while generating an immunostimulatory tumor microenvironment, linking suppression of antioxidant defense with immune remodeling (103). Similarly, the Fe-EGCG@RSL3 platform combined ferroptosis induction with TIME remodeling in bladder cancer (104). Targeted control can also be achieved at the level of iron availability. A ferritin-targeted biohybrid activated endogenous iron while replenishing exogenous iron to trigger ferroptosis-based immunotherapy, illustrating how delivery systems can spatially coordinate a fundamental biochemical requirement for ferroptosis (105).

Taken together, these platforms address different components of the same therapeutic problem: ferroptosis must be concentrated within the tumor, directed toward a relevant redox vulnerability and coordinated with immune activation. Thus, the major advantage of nanodelivery is potentially not greater ferroptosis per se, but greater spatial and mechanistic control over where and how ferroptosis occurs. Importantly, the therapeutic evidence for these ferroptosis-oriented nanoplatforms remains predominantly preclinical. Most reported benefits have been demonstrated in cell-based systems and mouse tumor models, whereas patient-associated evidence is largely correlative and does not establish clinical efficacy. Translation will require explicit characterization of pharmacokinetics, tissue distribution, tumor accumulation, systemic clearance and off-target exposure. Nanoparticle accumulation observed in experimental tumors may not be reproduced uniformly in patients because vascular permeability, stromal architecture and the enhanced permeability and retention effect vary substantially across tumors. In addition, prolonged retention of non-biodegradable or metal-containing materials in the liver, spleen or other organs may create systemic or long-term toxicities that are insufficiently captured by short-term tumor-growth studies (106, 107). Thus, improved antitumor efficacy in preclinical models should not by itself be interpreted as evidence of clinical readiness.

### 6.3 Microenvironmental remodeling and multimodal amplification

Ferroptosis-based combinations can extend beyond direct tumor-cell killing by simultaneously remodeling stromal or immune components of the tumor microenvironment. A dual-targeting strategy inhibited colorectal cancer liver metastasis through tumor-cell ferroptosis together with cancer-associated fibroblast reprogramming (108). Nanoparticle-modified nanofibers produced ferroptosis-associated oxidative and lipid-peroxidation changes alongside apoptosis and stimulated antitumor immunity in melanoma (109). These approaches suggest that ferroptosis may be more effective when oxidative tumor-cell damage is coupled to correction of the surrounding microenvironment rather than treated as an isolated cell-death event.

A second strategy is to exploit metabolic or pharmacological vulnerabilities that lower the threshold for ferroptosis while simultaneously supporting immunotherapy. Propafenone promoted mitochondria-associated ferroptosis and synergized with immunotherapy in melanoma (110), whereas simvastatin simultaneously inhibited PD-L1 through ILF3 and induced ferroptosis in gastric cancer cells (111). Although these interventions act through different molecular routes, they illustrate a common principle: agents with metabolic or signaling effects may enhance immunotherapy when they simultaneously weaken ferroptosis-defense pathways or checkpoint-associated immune escape.

Tumor-microenvironment-responsive Fe3+-doped nanocomposites generated iron-dependent oxidative stress, GSH depletion and lipid peroxidation consistent with a ferroptosis-associated response while enhancing immunotherapy (112), whereas two independent platforms combined ferroptosis with cuproptosis to potentiate immunotherapy (113, 114).

An HSA-templated platform enhanced radiosensitization, with inhibitor-rescue experiments indicating that both ferroptosis and apoptosis contributed to treatment-induced cell death (115), while transformable supramolecular peptides generated cascade self-enhanced ferroptosis and primed antitumor immunity (116). Collectively, these approaches suggest that the rationale for multimodal therapy should lie in mechanistic complementarity rather than simply increasing the number of cytotoxic modalities.

### 6.4 Principles for rational ferroptosis–immunotherapy combinations

The strongest therapeutic principle is selectivity. A useful ferroptosis-immunotherapy combination should increase ferroptosis in tumor cells or suppressive myeloid cells, preserve dendritic cells and CD8+ T cells, and include biomarkers that confirm on-target lipid peroxidation in the tumor. More broadly, emerging immune-checkpoint strategies increasingly emphasize combinations that engage distinct immune-cell populations and complementary mechanisms rather than relying on PD-1/PD-L1 blockade alone (117). This principle is particularly relevant to ferroptosis-based combinations, in which checkpoint selection should be aligned with the immune compartment and microenvironmental mechanism targeted by ferroptotic intervention. This design logic also explains why ferroptosis induction should be paired with myeloid blockade, checkpoint inhibition, immune agonists or targeted delivery rather than used as a blunt systemic stressor. At present, these principles should be regarded as design criteria for preclinical development rather than clinically validated treatment rules.

## 7 Biomarkers and translational barriers

Clinical translation requires tools that show where, when and in which cell type ferroptosis occurs. Without this information, ferroptosis induction cannot be separated from general oxidative damage, and therapeutic benefit cannot be distinguished from immune-cell toxicity.

### 7.1 Verifying and monitoring ferroptosis *in vivo*

An effective ferroptosis biomarker should answer more than whether lipid oxidation has occurred. Ideally, it should define the anatomical location of ferroptotic stress, identify the relevant cellular compartment and capture mechanisms that determine treatment sensitivity or resistance. Current approaches address these requirements at different levels. Importantly, detection of lipid oxidation or altered GPX4/SLC7A11 expression *in vivo* should be regarded as a ferroptosis-associated readout rather than definitive evidence of ferroptotic cell death unless supported by functional, pharmacological or genetic validation. Targeted positron-emission tomography-tracked biomimetic co-delivery amplified ferroptosis and pyroptosis while improving anti-PD-L1 efficacy in lung cancer models, illustrating how imaging can be incorporated into therapeutic design (118). Reviews of nanozymes for ferroptosis-based cancer theranostics support the broader idea that catalytic nanomaterials may combine ferroptosis induction, monitoring and treatment response assessment (119). Chemical design of magnetic nanomaterials for imaging and ferroptosis-based cancer therapy further shows that imaging-compatible ferroptosis tools are becoming a distinct translational area (120). These imaging-oriented approaches primarily address the first requirement—detecting and localizing ferroptosis-associated changes *in vivo*—but they do not by themselves establish which cellular population is undergoing ferroptosis or whether the observed oxidative state predicts treatment sensitivity.

Spatial profiling and functional genomics address a complementary limitation by providing information about the biological state underlying ferroptosis sensitivity or resistance. Monitoring should also capture resistance biology, not merely lipid oxidation intensity. Atlas-style analysis of metastatic gastric cancer linked ferroptosis to disease progression and immunotherapy response, supporting the use of spatial or single-cell approaches to connect ferroptosis states with treatment-relevant tissue organization (121). A CRISPR/Cas9 screen identified TRIM34 targeting as a way to enhance ferroptosis sensitivity and augment immunotherapy efficacy in hepatocellular carcinoma, illustrating how functional genomics can nominate biomarkers that are also intervention points (122). These approaches move the field toward actionable ferroptosis readouts rather than descriptive pathway scores.

Together, imaging, spatial profiling and functional screening therefore provide complementary rather than interchangeable information: imaging can localize treatment-associated changes, whereas molecular and functional approaches may help identify the mechanisms that determine whether those changes translate into therapeutic response.

### 7.2 Tumor-selective targeting and immune-cell protection

Achieving tumor selectivity is a prerequisite for immune-cell protection because systemic ferroptotic stress may injure beneficial immune populations. Targeted delivery strategies therefore provide one route for widening the therapeutic window of ferroptosis induction. A PFKFB4-targeting biomimetic co-delivery system promoted ferroptosis-associated cell death in small cell lung cancer and augmented anti-PD-L1 immunotherapy; notably, ferrostatin-1 only partially rescued the antiproliferative effect, indicating that ferroptosis contributed to, but did not fully account for, treatment-induced cell death (123). A bispecific CDH17-GUCY2C antibody-drug conjugate carrying the ferroptosis inducer RSL3 was developed for colorectal cancer, showing how lineage-restricted targeting may improve the therapeutic window of ferroptosis induction (124). A biomaterials study reported cell-specific ferroptosis targeting that killed tumors while sparing immune cells, providing a design principle for immune-preserving ferroptosis therapy (125). Reviews of ferroptosis in tumor-associated immune cells describe the process as a double-edged sword, because immune-cell ferroptosis can either relieve suppression or weaken antitumor immunity depending on the target population (126). These findings argue for delivery systems and biomarkers that distinguish malignant cells, suppressive myeloid cells and effector lymphocytes. Accordingly, a clinically useful ferroptosis readout should incorporate cellular identity rather than quantify lipid peroxidation alone, because the same biochemical event may indicate therapeutic tumor killing or harmful immune-cell injury depending on its cellular origin. From a translational perspective, immune-cell protection should therefore be evaluated as a formal safety endpoint rather than inferred from reduced tumor growth or the absence of overt systemic toxicity. Preclinical studies should assess whether ferroptosis-inducing formulations alter circulating and intratumoral CD8+ T cells, dendritic cells and other beneficial immune populations, together with conventional organ toxicity. Dose, exposure duration and tissue distribution are particularly important because a formulation that is tumor-selective at one exposure level may become immunotoxic when systemic exposure increases. These considerations argue for immune-subset-resolved toxicology and pharmacodynamic monitoring before ferroptosis-based combinations are advanced toward clinical testing.

### 7.3 Stratifying ferroptosis susceptibility and therapeutic resistance

Ferroptosis biomarkers should move beyond single-gene expression and integrate tumor lipid metabolism, GPX4/SLC7A11/FSP1/DHODH state, myeloid infiltration, CD8+ T-cell lipid stress, hypoxia and prior checkpoint exposure. A complementary therapeutic strategy is to identify tumor-intrinsic determinants of ferroptosis resistance that can serve as biomarkers or intervention points. Ferroptosis-associated molecular signatures have been linked to immune profiles and checkpoint responses in head and neck cancer (127). Reviews of metabolic regulation in breast cancer highlight ferroptosis as a convergence point for lipid metabolism, antioxidant defense and therapeutic sensitivity, supporting the broader relevance of metabolic patient stratification (128). Glioma studies linking ferroptosis-enriched cell-death programs to immunosuppression and immunotherapy resistance support the need for microenvironment-driven patient stratification (129). Dihydroorotate dehydrogenase has been reviewed as a mitochondrial ferroptosis regulator with therapeutic relevance, making mitochondrial defense a candidate axis for stratification and target selection (130). The next translational step is to connect these axes to prospective treatment decisions rather than retrospective molecular associations. Patient stratification should therefore integrate two dimensions: tumor susceptibility to ferroptosis and vulnerability of the surrounding immune compartment to ferroptotic injury. Such a framework may be more informative than single-gene ferroptosis scores alone.

## 8 Conclusion and outlook

Ferroptosis provides a compelling link between tumor-cell metabolism and immune remodeling. Its biochemical core is well defined by iron dependence, oxidizable phospholipids and antioxidant failure, but its immunological meaning depends on which cells experience lipid peroxidation and which cells receive the resulting signals. The most productive interpretation is therefore not that ferroptosis is simply immunogenic or suppressive, but that it is a context-dependent communication process within the TIME. Accordingly, we propose that the immunological consequences of ferroptosis should be interpreted along three interconnected dimensions: temporal pattern, spatial distribution and cellular target.

A synthesis of the available evidence suggests three provisional principles. First, tumor-cell ferroptosis can support antitumor immunity when it increases danger signaling, antigen presentation and sensitivity to CD8+ T-cell-derived interferon-gamma. Second, chronic lipid stress can weaken antitumor immunity by promoting suppressive myeloid states or damaging CD8+ T cells. Importantly, the proposed transition from acute, spatially restricted ferroptotic stress to chronic or broadly distributed immunosuppressive lipid stress has not yet been directly demonstrated in comparable longitudinal models and should therefore be regarded as a working hypothesis. Third, ferroptosis-based therapy is most rational when oxidative injury is spatially targeted, mechanistically paired with checkpoint blockade and monitored with biomarkers that distinguish tumor ferroptosis from immune-cell toxicity.

Several questions now define the field. The molecular boundary between immunogenic ferroptosis and inflammatory but suppressive ferroptosis remains incompletely resolved. TAMs, MDSCs and neutrophils require cell-type-specific models because their ferroptosis sensitivity can produce opposite immune effects. CD8+ T-cell protection must become a formal design criterion rather than an afterthought in ferroptosis-inducing therapy. Biomarker development should prioritize dynamic lipid-peroxidation imaging, spatial multi-omics and paired immune profiling so that ferroptosis can be measured in living tumors rather than inferred from static pathway scores.

Future ferroptosis-immunotherapy studies should therefore combine tumor-selective induction, immune-cell preservation, myeloid-state control and checkpoint blockade in models that retain spatial and metabolic features of human tumors. If these requirements are met, ferroptosis could become a strategic immune adjuvant for next-generation cancer therapy rather than another nonspecific cytotoxic stress.

## References

[1] Bagchi S Yuan R Engleman EG . Immune checkpoint inhibitors for the treatment of cancer: Clinical impact and mechanisms of response and resistance. Annu Rev Pathol. (2021) 16:223–49. doi: 10.1146/annurev-pathol-042020-042741 33197221

[2] Zheng Q Xu J Gu X Wu F Deng J Cai X . Immune checkpoint targeting TIGIT in hepatocellular carcinoma. Am J Trans Res. (2020) 12:3212–24. PMC7407725 32774695

[3] Vesely MD Zhang T Chen L . Resistance mechanisms to anti-PD cancer immunotherapy. Annu Rev Immunol. (2022) 40:45–74. doi: 10.1146/annurev-immunol-070621-030155 35471840

[4] Bell HN Zou W . Beyond the barrier: Unraveling the mechanisms of immunotherapy resistance. Annu Rev Immunol. (2024) 42:521–50. doi: 10.1146/annurev-immunol-101819-024752 38382538 PMC11213679

[5] Mandal K Barik GK Santra MK . Overcoming resistance to anti-PD-L1 immunotherapy: Mechanisms, combination strategies, and future directions. Mol Cancer. (2025) 24:246. doi: 10.1186/s12943-025-02400-z 41057853 PMC12505684

[6] Yi M Niu M Xu L Luo S Wu K . Regulation of PD-L1 expression in the tumor microenvironment. J Hematol Oncol. (2021) 14:10. doi: 10.1186/s13045-020-01027-5 33413496 PMC7792099

[7] Zhang H Liu L Liu J Dang P Hu S Yuan W . Roles of tumor-associated macrophages in anti-PD-1/PD-L1 immunotherapy for solid cancers. Mol Cancer. (2023) 22:58. doi: 10.1186/s12943-023-01725-x 36941614 PMC10029244

[8] Yang M Cui M Sun Y Liu S Jiang W . Mechanisms, combination therapy, and biomarkers in cancer immunotherapy resistance. Cell Comm Signaling CCS. (2024) 22:338. doi: 10.1186/s12964-024-01711-w 38898505 PMC11186190

[9] Kui Y Tong F Zhang R Dong X Wang J . Strategies to target PD-1/PD-L1 in the tumor microenvironment. Cell Oncol (Dordrecht Netherlands). (2026) 49:50. doi: 10.1007/s13402-026-01181-x 41784760 PMC12963217

[10] Topalian SL Forde PM Emens LA Yarchoan M Smith KN Pardoll DM . Neoadjuvant immune checkpoint blockade: A window of opportunity to advance cancer immunotherapy. Cancer Cell. (2023) 41:1551–66. doi: 10.1016/j.ccell.2023.07.011 37595586 PMC10548441

[11] Mortezaee K Majidpoor J Najafi S Tasa D . Bypassing anti-PD-(L)1 therapy: Mechanisms and management strategies. Biomedicine Pharmacotherapy = Biomedecine Pharmacotherapie. (2023) 158:114150. doi: 10.1016/j.biopha.2022.114150 36577330

[12] Dixon SJ Lemberg KM Lamprecht MR Skouta R Zaitsev EM Gleason CE . Ferroptosis: An iron-dependent form of nonapoptotic cell death. Cell. (2012) 149:1060–72. doi: 10.1016/j.cell.2012.03.042 22632970 PMC3367386

[13] Zhong H Liu H Fu Q . Ferroptosis as a therapeutic target in neurodegenerative diseases: Exploring the mechanisms and potential of treating Alzheimer's disease and Parkinson's disease. Protein Pept Lett. (2024) 31:759–72. doi: 10.2174/0109298665333926240927074528 39513303

[14] Yang WS SriRamaratnam R Welsch ME Shimada K Skouta R Viswanathan VS . Regulation of ferroptotic cancer cell death by GPX4. Cell. (2014) 156:317–31. doi: 10.1016/j.cell.2013.12.010 24439385 PMC4076414

[15] Doll S Proneth B Tyurina YY Panzilius E Kobayashi S Ingold I . ACSL4 dictates ferroptosis sensitivity by shaping cellular lipid composition. Nat Chem Biol. (2017) 13:91–8. doi: 10.1038/nchembio.2239 27842070 PMC5610546

[16] Zhang Y Zou L Li X Guo L Hu B Ye H . SLC40A1 in iron metabolism, ferroptosis, and disease: A review. WIREs Mech Dis. (2024) 16:e1644. doi: 10.1002/wsbm.1644 38508867

[17] Stockwell BR Friedmann Angeli JP Bayir H Bush AI Conrad M Dixon SJ . Ferroptosis: A regulated cell death nexus linking metabolism, redox biology, and disease. Cell. (2017) 171:273–85. doi: 10.1016/j.cell.2017.09.021 28985560 PMC5685180

[18] Xie Y Sun B Chen W Jia X Fan Y Xu Z . RNA modifications and cancer ferroptosis. Cancer Cell Int. (2026) 26:191. doi: 10.1186/s12935-026-04279-2 41904565 PMC13151117

[19] Lei G Zhuang L Gan B . The roles of ferroptosis in cancer: Tumor suppression, tumor microenvironment, and therapeutic interventions. Cancer Cell. (2024) 42:513–34. doi: 10.1016/j.ccell.2024.03.011 38593779

[20] Chen F Kang R Tang D Liu J . Ferroptosis: Principles and significance in health and disease. J Hematol Oncol. (2024) 17:41. doi: 10.1186/s13045-024-01564-3 38844964 PMC11157757

[21] Mishima E Nakamura T Doll S Proneth B Fedorova M Pratt DA . Recommendations for robust and reproducible research on ferroptosis. Nat Rev Mol Cell Biol. (2025) 26:615–30. doi: 10.1038/s41580-025-00843-2 40204928

[22] Galluzzi L Vitale I Aaronson SA Abrams JM Adam D Agostinis P . Molecular mechanisms of cell death: Recommendations of the nomenclature committee on cell death 2018. Cell Death Differ. (2018) 25:486–541. doi: 10.1038/s41418-017-0012-4 29362479 PMC5864239

[23] Xu H Ye D Ren M Zhang H Bi F . Ferroptosis in the tumor microenvironment: Perspectives for immunotherapy. Trends Mol Med. (2021) 27:856–67. doi: 10.1016/j.molmed.2021.06.014 34312075

[24] Mbah NE Lyssiotis CA . Metabolic regulation of ferroptosis in the tumor microenvironment. J Biol Chem. (2022) 298:101617. doi: 10.1016/j.jbc.2022.101617 35065965 PMC8892088

[25] Yang K Wang X Song C He Z Wang R Xu Y . The role of lipid metabolic reprogramming in tumor microenvironment. Theranostics. (2023) 13:1774–808. doi: 10.7150/thno.82920 37064872 PMC10091885

[26] Gong D Chen M Wang Y Shi J Hou Y . Role of ferroptosis on tumor progression and immunotherapy. Cell Death Discov. (2022) 8:427. doi: 10.1038/s41420-022-01218-8 36289191 PMC9605952

[27] Bhowmick S Banerjee S Shridhar V Mondal S . Reprogrammed immuno-metabolic environment of cancer: The driving force of ferroptosis resistance. Mol Cancer. (2025) 24:161. doi: 10.1186/s12943-025-02337-3 40462094 PMC12131403

[28] Tao Q Liu N Chen J Wu J Li J Chen X . Ferroptosis and tumor immunity. Zhong Nan Da Xue Xue Bao Yi Xue Ban = J Cent South Univ Med Sci. (2024) 49:1309–15. doi: 10.11817/j.issn.1672-7347.2024.240389 39788519 PMC11628223

[29] Yun T Liu Z Wang J Wang R Zhu L Zhu Z . Microenvironment immune response induced by tumor ferroptosis-the application of nanomedicine. Front Oncol. (2022) 12:1019654. doi: 10.3389/fonc.2022.1019654 36185311 PMC9523366

[30] Li J Liu J Zhou Z Wu R Chen X Yu C . Tumor-specific GPX4 degradation enhances ferroptosis-initiated antitumor immune response in mouse models of pancreatic cancer. Sci Transl Med. (2023) 15:eadg3049. doi: 10.1126/scitranslmed.adg3049 37910602

[31] Efimova I Catanzaro E Van der Meeren L Turubanova VD Hammad H Mishchenko TA . Vaccination with early ferroptotic cancer cells induces efficient antitumor immunity. J Immunother Cancer. (2020) 8:e001369. doi: 10.1136/jitc-2020-001369 33188036 PMC7668384

[32] Li G Liao C Chen J Wang Z Zhu S Lai J . Targeting the MCP-GPX4/HMGB1 axis for effectively triggering immunogenic ferroptosis in pancreatic ductal adenocarcinoma. Advanced Sci (Weinheim Baden-Wurttemberg Germany). (2024) 11:e2308208. doi: 10.1002/advs.202308208 38593415 PMC11151063

[33] Saviuk M Turubanova VD De Brée S Van Lint S Maia TM Devos S . Ferroptosis-armed dendritic cell vaccines for glioma immunotherapy. Nat Commun. (2026) 17:6158. doi: 10.1038/s41467-026-72737-6 42098128 PMC13365579

[34] Deng Z Li B Yang M Lu L Shi X Lovell JF . Irradiated microparticles suppress prostate cancer by tumor microenvironment reprogramming and ferroptosis. J Nanobiotechnol. (2024) 22:225. doi: 10.1186/s12951-024-02496-3 38705987 PMC11070086

[35] Guo Y Li Y Zhang M Ma R Wang Y Weng X . Polymeric nanocarrier via metabolism regulation mediates immunogenic cell death with spatiotemporal orchestration for cancer immunotherapy. Nat Commun. (2024) 15:8586. doi: 10.1038/s41467-024-53010-0 39362879 PMC11450208

[36] Liao H Chen M Liao Z Luo Y Chen S Tan W . Sonodynamic therapy-boosted biomimetic nanoplatform targets ferroptosis and CD47 as vulnerabilities for cancer immunotherapy. J Nanobiotechnol. (2025) 23:432. doi: 10.1186/s12951-025-03485-w 40490790 PMC12150583

[37] Li Y Chen J Xia Q Shang J He Y Li Z . Photothermal Fe(3)O(4) nanoparticles induced immunogenic ferroptosis for synergistic colorectal cancer therapy. J Nanobiotechnol. (2024) 22:630. doi: 10.1186/s12951-024-02909-3 39415226 PMC11484360

[38] Ma C Hu H Liu H Zhong C Wu B Lv C . Lipotoxicity, lipid peroxidation and ferroptosis: A dilemma in cancer therapy. Cell Biol Toxicol. (2025) 41:75. doi: 10.1007/s10565-025-10025-7 40285867 PMC12033115

[39] Catanzaro E Demuynck R Naessens F Galluzzi L Krysko DV . Immunogenicity of ferroptosis in cancer: A matter of context? Trends Cancer. (2024) 10:407–16. doi: 10.1016/j.trecan.2024.01.013 38368244

[40] Tang D Kroemer G Kang R . Ferroptosis in immunostimulation and immunosuppression. Immunol Rev. (2024) 321:199–210. doi: 10.1111/imr.13235 37424139

[41] Demuynck R Efimova I Naessens F Krysko DV . Immunogenic ferroptosis and where to find it? J Immunother Cancer. (2021) 9:e003430. doi: 10.1136/jitc-2021-003430 34903554 PMC8671998

[42] Tang R Xu J Zhang B Liu J Liang C Hua J . Ferroptosis, necroptosis, and pyroptosis in anticancer immunity. J Hematol Oncol. (2020) 13:110. doi: 10.1186/s13045-020-00946-7 32778143 PMC7418434

[43] He N Yuan D Luo M Xu Q Wen Z Wang Z . Ferroptosis contributes to immunosuppression. Front Med. (2025) 19:1–22. doi: 10.1007/s11684-024-1080-8 39560919

[44] Lan J Cai D Gou S Bai Y Lei H Li Y . The dynamic role of ferroptosis in cancer immunoediting: Implications for immunotherapy. Pharmacol Res. (2025) 214:107674. doi: 10.1016/j.phrs.2025.107674 40020885

[45] Wiernicki B Maschalidi S Pinney J Adjemian S Vanden Berghe T Ravichandran KS . Cancer cells dying from ferroptosis impede dendritic cell-mediated anti-tumor immunity. Nat Commun. (2022) 13:3676. doi: 10.1038/s41467-022-31218-2 35760796 PMC9237053

[46] Xiao K Zhang S Peng Q Du Y Yao X Ng II . PD-L1 protects tumor-associated dendritic cells from ferroptosis during immunogenic chemotherapy. Cell Rep. (2024) 43:114868. doi: 10.1016/j.celrep.2024.114868 39423128

[47] Kim R Taylor D Vonderheide RH Gabrilovich DI . Ferroptosis of immune cells in the tumor microenvironment. Trends Pharmacol Sci. (2023) 44:542–52. doi: 10.1016/j.tips.2023.06.005 37380530

[48] Zhu W Liu X Yang L He Q Huang D Tan X . Ferroptosis and tumor immunity: In perspective of the major cell components in the tumor microenvironment. Eur J Pharmacol. (2023) 961:176124. doi: 10.1016/j.ejphar.2023.176124 37925133

[49] Qi Y Li Q Chen L Zhao S Nie J Liu G . A new perspective: Acyl-CoA synthetase long-chain family member 4 inhibits ubiquitin-specific protease 7-induced epithelial ovarian cancer progression by inducing ferroptosis and M1 macrophage polarization. Cytojournal. (2025) 22:28. doi: 10.25259/Cytojournal_241_2024 40260070 PMC12010880

[50] Wang Z Xu W Zhao L Zhong L Yu W Wang S . IL4I1^+^ macrophages and TDO2^+^ myofibroblasts drive AhR-mediated immunosuppression and ferroptosis resistance in solid predominant lung adenocarcinoma. Advanced Sci (Weinheim Baden-Wurttemberg Germany). (2026) 13:e13606. doi: 10.1002/advs.202513606 41431173 PMC12955909

[51] Wang S Zhu L Li T Lin X Zheng Y Xu D . Disruption of MerTK increases the efficacy of checkpoint inhibitor by enhancing ferroptosis and immune response in hepatocellular carcinoma. Cell Rep Med. (2024) 5:101415. doi: 10.1016/j.xcrm.2024.101415 38382467 PMC10897610

[52] Tang C He C Wang D Guo J Yin X Ye H . Co-delivery of sorafenib and an FSP1 inhibitor triggers dual ferroptosis in tumor cells and immunosuppressive macrophages for enhanced immunotherapy in mouse models of hepatocellular carcinoma. Nat Commun. (2025) 16:10096. doi: 10.1038/s41467-025-65056-9 41253833 PMC12627561

[53] Hao X Zheng Z Liu H Zhang Y Kang J Kong X . Inhibition of APOC1 promotes the transformation of M2 into M1 macrophages via the ferroptosis pathway and enhances anti-PD1 immunotherapy in hepatocellular carcinoma based on single-cell RNA sequencing. Redox Biol. (2022) 56:102463. doi: 10.1016/j.redox.2022.102463 36108528 PMC9482117

[54] Lin H Tison K Du Y Kirchhoff P Kim C Wang W . Itaconate transporter SLC13A3 impairs tumor immunity via endowing ferroptosis resistance. Cancer Cell. (2024) 42:2032–2044.e2036. doi: 10.1016/j.ccell.2024.10.010 39515327 PMC11631639

[55] Jiang Z Lim SO Yan M Hsu JL Yao J Wei Y . TYRO3 induces anti-PD-1/PD-L1 therapy resistance by limiting innate immunity and tumoral ferroptosis. J Clin Invest. (2021) 131:e139434. doi: 10.1172/jci139434 33855973 PMC8262501

[56] Sha X Wang C Liu Y Zhong N Lu Y Zhang Q . Multifunctional glycyrrhizic acid-loaded nanoplatform combining ferroptosis induction and HMGB1 blockade for enhanced tumor immunotherapy. J Nanobiotechnol. (2025) 23:224. doi: 10.1186/s12951-025-03307-z 40108690 PMC11924601

[57] Zhao YY Lian JX Lan Z Zou KL Wang WM Yu G T . Ferroptosis promotes anti-tumor immune response by inducing immunogenic exposure in HNSCC. Oral Dis. (2023) 29:933–41. doi: 10.1111/odi.14077 34773344

[58] Li N Song X Peng X Li M Zhu M Xiao R . Tim-3 facilitates dendritic cell ferroptosis and impairs antitumor immunity in steatohepatitis-related HCC. J Hepatol. (2026) 85:331–44. doi: 10.1016/j.jhep.2026.04.010 42035954

[59] Conche C Finkelmeier F Pešić M Nicolas AM Böttger TW Kennel KB . Combining ferroptosis induction with MDSC blockade renders primary tumours and metastases in liver sensitive to immune checkpoint blockade. Gut. (2023) 72:1774–82. doi: 10.1136/gutjnl-2022-327909 36707233 PMC10423492

[60] Wang Y Zhang X Yang W Liu X Xie S Zhang L . Caveolin-1 drives ferroptosis in MDSCs via PKA-DRP1-mediated ER–mitochondria crosstalk to shape breast cancer immunosuppression. Free Radical Biol Med. (2026) 242:601–13. doi: 10.1016/j.freeradbiomed.2025.11.001 41205773

[61] Mohamady Farouk Abdalsalam N Liang Z Kashaf Tariq H Ibrahim A Li R Wan X . Etomoxir sodium salt promotes imidazole ketone erastin-induced myeloid-derived suppressor cell ferroptosis and enhances cancer therapy. Biology. (2024) 13:949. doi: 10.3390/biology13110949 39596904 PMC11592117

[62] Zhao Y Liu Z Liu G Zhang Y Liu S Gan D . Neutrophils resist ferroptosis and promote breast cancer metastasis through aconitate decarboxylase 1. Cell Metab. (2023) 35:1688–1703.e1610. doi: 10.1016/j.cmet.2023.09.004 37793345 PMC10558089

[63] Kim R Hashimoto A Markosyan N Tyurin VA Tyurina YY Kar G . Ferroptosis of tumour neutrophils causes immune suppression in cancer. Nature. (2022) 612:338–46. doi: 10.1038/s41586-022-05443-0 36385526 PMC9875862

[64] Zhu X Zheng W Wang X Li Z Shen X Chen Q . Enhanced photodynamic therapy synergizing with inhibition of tumor neutrophil ferroptosis boosts anti-PD-1 therapy of gastric cancer. Advanced Sci (Weinheim Baden-Wurttemberg Germany). (2024) 11:e2307870. doi: 10.1002/advs.202307870 38233204 PMC10966534

[65] Zheng N Li F Huang Q Huang X Maj T . Macrophages and macrophage extracellular vesicles confer cancer ferroptosis resistance via PRDX6-mediated mitophagy inhibition. Redox Biol. (2025) 86:103826. doi: 10.1016/j.redox.2025.103826 40825268 PMC12391288

[66] Luo J Zhang X Liang Z Zhuang W Jiang M Ma M . ISCU-p53 axis orchestrates macrophage polarization to dictate immunotherapy response in esophageal squamous cell carcinoma. Cell Death Dis. (2025) 16:462. doi: 10.1038/s41419-025-07787-7 40541964 PMC12181301

[67] Zhang J Jin X Abulaihaiti M Liu X Zeng L Xiao Y . Hypoxic tumor exosomes suppress macrophage inflammation and ferroptosis via NDUFV2 to enhance bystander tumor radioresistance. Cell Death Dis. (2025) 17:109. doi: 10.1038/s41419-025-08357-7 41419454 PMC12847814

[68] Zhu GQ Tang Z Huang R Qu WF Fang Y Yang R . CD36(+) cancer-associated fibroblasts provide immunosuppressive microenvironment for hepatocellular carcinoma via secretion of macrophage migration inhibitory factor. Cell Discov. (2023) 9:25. doi: 10.1038/s41421-023-00529-z 36878933 PMC9988869

[69] Wang K Xiao Y Ren J Zuo C Mu L Li Q . Ferroptosis and low-grade glioma: The breakthrough potential of NUAK2. Free Radical Biol Med. (2025) 234:203–19. doi: 10.1016/j.freeradbiomed.2025.04.035 40286882

[70] Wang W Green M Choi JE Gijón M Kennedy PD Johnson JK . CD8(+) T cells regulate tumour ferroptosis during cancer immunotherapy. Nature. (2019) 569:270–4. doi: 10.1038/s41586-019-1170-y 31043744 PMC6533917

[71] Liao P Wang W Wang W Kryczek I Li X Bian Y . CD8(+) T cells and fatty acids orchestrate tumor ferroptosis and immunity via ACSL4. Cancer Cell. (2022) 40:365–378.e366. doi: 10.1016/j.ccell.2022.02.003 35216678 PMC9007863

[72] Tao Q Liu N Wu J Chen J Chen X Peng C . Mefloquine enhances the efficacy of anti-PD-1 immunotherapy via IFN-γ-STAT1-IRF1-LPCAT3-induced ferroptosis in tumors. J Immunother Cancer. (2024) 12:e008554. doi: 10.1136/jitc-2023-008554 38471712 PMC10936479

[73] Liu C Hu C Cheng J Xin D Jin S Tian W . AGPAT3 reshapes tumor cell vulnerability to IFNγ-mediated ferroptosis and enhances immunotherapy efficacy through lipid remodeling. J Immunother Cancer. (2026) 14:e013305. doi: 10.1136/jitc-2025-013305 41807033 PMC12983827

[74] Li H Sun Y Yao Y Ke S Zhang N Xiong W . USP8-governed GPX4 homeostasis orchestrates ferroptosis and cancer immunotherapy. PNAS. (2024) 121:e2315541121. doi: 10.1073/pnas.2315541121 38598341 PMC11032464

[75] Zheng P Hu Z Shen Y Gu L Ouyang Y Duan Y . PSAT1 impairs ferroptosis and reduces immunotherapy efficacy via GPX4 hydroxylation. Nat Chem Biol. (2025) 21:1420–32. doi: 10.1038/s41589-025-01887-3 40281343

[76] Freitas-Cortez MA Masrorpour F Jiang H Mahmud I Lu Y Huang A . Cancer cells avoid ferroptosis induced by immune cells via fatty acid binding proteins. Mol Cancer. (2025) 24:40. doi: 10.1186/s12943-024-02198-2 39901247 PMC11789333

[77] Alizadeh H Kerachian S Babashah S Soltani B M . Introduction of AGPAT3 gene as a regulator of cisplatin resistance in A2780 ovarian endometrioid carcinoma cell line. PloS One. (2025) 20:e0318740. doi: 10.1371/journal.pone.0318740 40063560 PMC11892817

[78] Corno C D'Arcy P Bagnoli M Paolini B Costantino M Carenini N . The deubiquitinase USP8 regulates ovarian cancer cell response to cisplatin by suppressing apoptosis. Front Cell Dev Biol. (2022) 10:1055067. doi: 10.3389/fcell.2022.1055067 36578788 PMC9791127

[79] Luo MY Zhou Y Gu WM Wang C Shen NX Dong JK . Metabolic and nonmetabolic functions of PSAT1 coordinate signaling cascades to confer EGFR inhibitor resistance and drive progression in lung adenocarcinoma. Cancer Res. (2022) 82:3516–31. doi: 10.1158/0008-5472.Can-21-4074 36193649

[80] Lv Y Tang W Xu Y Chang W Zhang Z Lin Q . Apolipoprotein L3 enhances CD8+ T cell antitumor immunity of colorectal cancer by promoting LDHA-mediated ferroptosis. Int J Biol Sci. (2023) 19:1284–98. doi: 10.7150/ijbs.74985 36923931 PMC10008698

[81] Li S Shapiro JW Shah H Higgs EF Xie L Li Y . Tumor cell-intrinsic Decr2 regulates ferroptosis and immunotherapy efficacy. Cancer Immunol Res. (2025) 13:1284–302. doi: 10.1158/2326-6066.Cir-24-0519 40512146 PMC12314507

[82] Ma X Xiao L Liu L Ye L Su P Bi E . CD36-mediated ferroptosis dampens intratumoral CD8(+) T cell effector function and impairs their antitumor ability. Cell Metab. (2021) 33:1001–1012.e1005. doi: 10.1016/j.cmet.2021.02.015 33691090 PMC8102368

[83] Xiang B Zhang M Li K Zhang Z Liu Y Gao M . The epitranscriptional factor PCIF1 orchestrates CD8(+) T cell ferroptosis and activation to control antitumor immunity. Nat Immunol. (2025) 26:252–64. doi: 10.1038/s41590-024-02047-w 39762445

[84] Xu C Sun S Johnson T Qi R Zhang S Zhang J . The glutathione peroxidase Gpx4 prevents lipid peroxidation and ferroptosis to sustain Treg cell activation and suppression of antitumor immunity. Cell Rep. (2021) 35:109235. doi: 10.1016/j.celrep.2021.109235 34133924

[85] Yang T Liu Z Fu Z Zhang X Cao Y Liang Q . Albumin nanoassembly bi-directionally manipulated ferroptosis in tumor and CD8(+) T cells for triple-negative breast cancer therapy. Asian J Pharm Sci. (2025) 20:100970. doi: 10.1016/j.ajps.2024.100970 40213381 PMC11985007

[86] Xue Y Lu F Chang Z Li J Gao Y Zhou J . Intermittent dietary methionine deprivation facilitates tumoral ferroptosis and synergizes with checkpoint blockade. Nat Commun. (2023) 14:4758. doi: 10.1038/s41467-023-40518-0 37553341 PMC10409767

[87] Tzeng SF Yu YR Park J von Renesse J Hsiao HW Hsu CH . PLT012, a humanized CD36-blocking antibody, is effective for unleashing antitumor immunity against liver cancer and liver metastasis. Cancer Discov. (2025) 15:1676–96. doi: 10.1158/2159-8290.Cd-24-1409 40294022 PMC7617665

[88] Haoyue W Kexiang S Shan TW Jiamin G Luyun Y Junkai W . Icariin promoted ferroptosis by activating mitochondrial dysfunction to inhibit colorectal cancer and synergistically enhanced the efficacy of PD-1 inhibitors. Phytomedicine Int J Phytotherapy Phytopharmacology. (2025) 136:156224. doi: 10.1016/j.phymed.2024.156224 39642461

[89] Yu T Zhao X Dong C Lu H Wang Y Luo X . AZ-628 sensitizes donafenib in hepatocellular carcinoma by targeting tyrosine kinase pathway and ferroptosis. Cytojournal. (2025) 22:88. doi: 10.25259/Cytojournal_273_2024 41312539 PMC12653991

[90] Ma L Chen C Zhao C Li T Ma L Jiang J . Targeting carnitine palmitoyl transferase 1A (CPT1A) induces ferroptosis and synergizes with immunotherapy in lung cancer. Signal Transduction Targeted Ther. (2024) 9:64. doi: 10.1038/s41392-024-01772-w 38453925 PMC10920667

[91] Chen C Yang Y Guo Y He J Chen Z Qiu S . CYP1B1 inhibits ferroptosis and induces anti-PD-1 resistance by degrading ACSL4 in colorectal cancer. Cell Death Dis. (2023) 14:271. doi: 10.1038/s41419-023-05803-2 37059712 PMC10104818

[92] Moradi Kashkooli F Mirala F Tehrani HH Alirahimi M Souri M Golzaryan A . Mechanical forces in tumor growth and treatment: Perspectives from biology, physics, engineering, and mathematical modeling. WIREs Mech Dis. (2025) 17:e70000. doi: 10.1002/wsbm.70000 40170456

[93] Wang S Yuan X Yang Z Zhang X Xu Z Yang L . Matrix stiffness-dependent PD-L2 deficiency improves SMYD3/xCT-mediated ferroptosis and the efficacy of anti-PD-1 in HCC. J Adv Res. (2025) 73:265–82. doi: 10.1016/j.jare.2024.08.021 39159723 PMC12225897

[94] Li D Dian Y Li L Du S Meng Y Yao L . Kitasamycin overcomes ferroptosis and immunotherapy resistance by targeting the HUWE1-NCOA4-FTH1 axis. Autophagy. (2026) 22:1003–20. doi: 10.1080/15548627.2026.2623986 41612599 PMC13233000

[95] Liu X Fang S Ren X Hong Y Guo J Liu X . GBP2 condensates promote ferroptosis to sensitize anti-PD-L1 immunotherapy in melanoma. Nat Commun. (2025) 17:964. doi: 10.1038/s41467-025-67690-9 41444224 PMC12847934

[96] Zhou X Wang G Wu Y Wu M Zhai X Tian C . SLC6A8-mediated creatine uptake suppresses ERK2-FSP1 signaling and induces ferroptosis in colorectal cancer. Cell Rep. (2025) 44:116139. doi: 10.1016/j.celrep.2025.116139 40892544

[97] Hwang D Henry WS . Targeting GPX4 palmitoylation to boost antitumor immunity. Trends Cancer. (2025) 11:491–2. doi: 10.1016/j.trecan.2025.05.001 40374398

[98] He Z Zheng D Li F Chen L Wu C Zeng Z . TMOD3 accelerated resistance to immunotherapy in KRAS-mutated pancreatic cancer through promoting autophagy-dependent degradation of ASCL4. Drug Resist Updates Rev Commentaries Antimicrobial Anticancer Chemotherapy. (2025) 78:101171. doi: 10.1016/j.drup.2024.101171 39531951

[99] Zhou Q Sun Y Du S Dian Y Yao L Su H . Vorapaxar enhanced mitochondria-associated ferroptosis primes cancer immunotherapy via targeting FOXO1/HMOX1 axis. Cell Rep Med. (2025) 6:102371. doi: 10.1016/j.xcrm.2025.102371 41005298 PMC12629800

[100] Zhu J Xiong Y Zhang Y Liang H Cheng K Lu Y . Simvastatin overcomes the pPCK1-pLDHA-SPRINGlac axis-mediated ferroptosis and chemo-immunotherapy resistance in AKT-hyperactivated intrahepatic cholangiocarcinoma. Cancer Commun (London England). (2025) 45:1038–71. doi: 10.1002/cac2.70036 40443016 PMC12365544

[101] Huang H Ye F Liu T Hong J Jiang H Chen Z . Nano-immunotherapy synergizing ferroptosis and STING activation in metastatic bladder cancer. Cyborg Bionic Syst (Washington DC). (2026) 7:458. doi: 10.34133/cbsystems.0458 41522604 PMC12783508

[102] Ding Q Tang W Li X Ding Y Chen X Cao W . Mitochondrial-targeted brequinar liposome boosted mitochondrial-related ferroptosis for promoting checkpoint blockade immunotherapy in bladder cancer. J Controlled Release Off J Controlled Release Soc. (2023) 363:221–34. doi: 10.1016/j.jconrel.2023.09.024 37717657

[103] Hsieh CH Hsieh HC Shih FS Wang PW Yang LX Shieh DB . An innovative NRF2 nano-modulator induces lung cancer ferroptosis and elicits an immunostimulatory tumor microenvironment. Theranostics. (2021) 11:7072–91. doi: 10.7150/thno.57803 34093872 PMC8171079

[104] Zhang C Liu S Zhang J Lu J Chen Z Pan B . A multifunctional Fe-EGCG@RSL3 nanomedicine synergizes ferroptosis induction and tumor microenvironment remodeling for enhanced bladder cancer immunotherapy. Res (Washington DC). (2025) 8:735. doi: 10.34133/research.0735 40530387 PMC12173478

[105] Sheng S Zhang Y Jin L Sun W Zhu D Mei L . A ferritin-targeted biohybrid triggering ferroptosis immunotherapy via activating endogenous iron and replenishing exogenous iron simultaneously. Nat Commun. (2025) 16:6045. doi: 10.1038/s41467-025-61419-4 40593723 PMC12217129

[106] Wei Q Xu Y Zhang T Xu S Wang H Wang Y . Nano delivery strategy opens effective avenues for the treatment of colorectal cancer. Med Bull. (2026) 2:205–30. doi: 10.1002/mdb2.7002842587375

[107] Mohammadi T Gheybalizadeh H Rahimpour E Soleymani J Shafiei-Irannejad V . Advanced photoluminescent nanomaterials for targeted bioimaging of cancer cells. Heliyon. (2025) 11:e41566. doi: 10.1016/j.heliyon.2024.e41566 39850435 PMC11754178

[108] Wu Z Lin X Ying Y Fan G Shi J Zheng X . A dual-targeting strategy to inhibit colorectal cancer liver metastasis via tumor cell ferroptosis and cancer-associated fibroblast reprogramming. Bioact Mater. (2025) 52:73–91. doi: 10.1016/j.bioactmat.2025.05.025 40530415 PMC12173058

[109] Li M Li R Xu B Lian Y Yang C Li J . Nanoparticle-modified nanofibers induce ferroptosis and stimulate antitumor immunity for melanoma therapy. Advanced Sci (Weinheim Baden-Wurttemberg Germany). (2026) 13:e08753. doi: 10.1002/advs.202508753 41114460 PMC12767067

[110] Zhou Q Dian Y He Y Yao L Su H Meng Y . Propafenone facilitates mitochondrial-associated ferroptosis and synergizes with immunotherapy in melanoma. J Immunother Cancer. (2024) 12:e009805. doi: 10.1136/jitc-2024-009805 39581704 PMC11590812

[111] Sun D Cui X Yang W Wei M Yan Z Zhang M . Simvastatin inhibits PD-L1 via ILF3 to induce ferroptosis in gastric cancer cells. Cell Death Dis. (2025) 16:208. doi: 10.1038/s41419-025-07562-8 40140647 PMC11947124

[112] Feng L Sang J Zhu H Hu Y Liu B He G . Tumor microenvironment-activated Fe(3+)-doped dendritic mesoporous organosilica nanocomposites as ferroptosis inducers for enhanced immunotherapy. Advanced Mat (Deerfield Beach Fla). (2025) 37:e10010. doi: 10.1002/adma.202510010 40855985

[113] Chen T Huang C Liu Y Nie D Chen J . Bimetallic nanozyme-mediated dual ferroptosis/cuproptosis synergy potentiates immunotherapy in bladder cancer. Colloids Surfaces B Biointerfaces. (2025) 255:114954. doi: 10.1016/j.colsurfb.2025.114954 40706095

[114] Li Y Liu J Chen Y Weichselbaum RR Lin W . Nanoparticles synergize ferroptosis and cuproptosis to potentiate cancer immunotherapy. Advanced Sci (Weinheim Baden-Wurttemberg Germany). (2024) 11:e2310309. doi: 10.1002/advs.202310309 38477411 PMC11187894

[115] Fan X Liu J Xie S Tan H Mi Z Zhang P . HSA-templated synergistic platform boosts radiotherapy via enhanced radiosensitization and ferroptosis induction. Drug Resist Updates Rev Commentaries Antimicrobial Anticancer Chemotherapy. (2025) 83:101293. doi: 10.1016/j.drup.2025.101293 40882450

[116] Wang H Jiao D Feng D Liu Q Huang Y Hou J . Transformable supramolecular self-assembled peptides for cascade self-enhanced ferroptosis primed cancer immunotherapy. Advanced Mat (Deerfield Beach Fla). (2024) 36:e2311733. doi: 10.1002/adma.202311733 38339920

[117] Li Y Kang Z Du Y . Beyond PD-1/PD-L1: New immune checkpoints and therapeutic combinations in cancer immunotherapy. Med Bull. (2026) 2:61–93. doi: 10.1002/mdb2.7001842587375

[118] Zhu J Zhou W Yao Y Zhou X Ma X Zhang B . Targeted positron emission tomography-tracked biomimetic codelivery synergistically amplifies ferroptosis and pyroptosis for inducing lung cancer regression and anti-PD-L1 immunotherapy efficacy. ACS Nano. (2024) 18:31401–20. doi: 10.1021/acsnano.4c11278 39475541

[119] Khan RU Qian L Min J Peng H Wang F . Nanozymes for ferroptosis-based cancer theranostics. Bioact Mater. (2026) 62:862–82. doi: 10.1016/j.bioactmat.2026.03.043 42004619 PMC13089176

[120] Xu W Guan G Yue R Dong Z Lei L Kang H . Chemical design of magnetic nanomaterials for imaging and ferroptosis-based cancer therapy. Chem Rev. (2025) 125:1897–961. doi: 10.1021/acs.chemrev.4c00546 39951340

[121] Cheng X Dai E Wu J Flores NM Chu Y Wang R . Atlas of metastatic gastric cancer links ferroptosis to disease progression and immunotherapy response. Gastroenterology. (2024) 167:1345–57. doi: 10.1053/j.gastro.2024.07.038 39097198

[122] Yao F Zhou S Zhang R Chen Y Huang W Yu K . CRISPR/Cas9 screen reveals that targeting TRIM34 enhances ferroptosis sensitivity and augments immunotherapy efficacy in hepatocellular carcinoma. Cancer Lett. (2024) 593:216935. doi: 10.1016/j.canlet.2024.216935 38704136

[123] Liu X He J Ying H Chen C Zheng C Luo P . Targeting PFKFB4 biomimetic codelivery system synergistically enhances ferroptosis to suppress small cell lung cancer and augments the efficacy of anti-PD-L1 immunotherapy. Advanced Sci (Weinheim Baden-Wurttemberg Germany). (2025) 12:e2417374. doi: 10.1002/advs.202417374 40213972 PMC12165097

[124] Zhang Y Du J Cui X Ling Y Tang C . Development of a bispecific CDH17-GUCY2C ADC bearing the ferroptosis inducer RSL3 for the treatment of colorectal cancer. Cell Death Discov. (2025) 11:347. doi: 10.1038/s41420-025-02652-0 40721409 PMC12304242

[125] Wang Y Han Y Li J Wang Z Li K Bai T . Cell-specific ferroptosis targeting tumors while sparing immune cells. Biomaterials. (2025) 323:123457. doi: 10.1016/j.biomaterials.2025.123457 40460787

[126] Hu J Cui L Hou B Ding X Liu H Sun W . Ferroptosis in tumor associated immune cells: A double-edged sword against tumors. Crit Rev Oncology/Hematol. (2025) 212:104818. doi: 10.1016/j.critrevonc.2025.104818 40570995

[127] Chung CH Lin CY Chen CY Hsueh CW Chang YW Wang CC . Ferroptosis signature shapes the immune profiles to enhance the response to immune checkpoint inhibitors in head and neck cancer. Advanced Sci (Weinheim Baden-Wurttemberg Germany). (2023) 10:e2204514. doi: 10.1002/advs.202204514 37026630 PMC10214241

[128] Glibetic N Weichhaus M . Metabolic regulation of ferroptosis in breast cancer. Int J Mol Sci. (2025) 26:9686. doi: 10.3390/ijms26199686 41096949 PMC12524448

[129] Liu T Zhu C Chen X Guan G Zou C Shen S . Ferroptosis, as the most enriched programmed cell death process in glioma, induces immunosuppression and immunotherapy resistance. Neuro-Oncology. (2022) 24:1113–25. doi: 10.1093/neuonc/noac033 35148413 PMC9248406

[130] Lee J Roh JL . Dihydroorotate dehydrogenase in mitochondrial ferroptosis and cancer therapy. Cells. (2025) 14:1889. doi: 10.3390/cells14231889 41369379 PMC12691135

